# Genome-wide association study and high-quality gene mining related to soybean protein and fat

**DOI:** 10.1186/s12864-023-09687-6

**Published:** 2023-10-07

**Authors:** Qi Zhang, Tingting Sun, Jiabao Wang, JianBo Fei, Yufu Liu, Lu Liu, Peiwu Wang

**Affiliations:** 1 Jilin Agricultural University, Changchun, China; 2 JiLin Agricultural Science and Technology University, Jilin, China; 3 Jilin Provincial Seed Management Station, Jilin, China

**Keywords:** Soybean, SLAF-seq, Genome-Wide Association Study, Protein; Fat

## Abstract

**Background:**

Soybean is one of the most important oil crops in the world, and its protein and fat are the primary sources of edible oil and vegetable protein. The effective components in soybean protein and fat have positive effects on improving human immunity, anti-tumor, and regulating blood lipids and metabolism. Therefore, increasing the contents of protein and fat in soybeans is essential for improving the quality of soybeans.

**Results:**

This study selected 292 soybean lines from different regions as experimental materials, based on SLAF-seq sequencing technology, and performed genome-wide association study (GWAS) on the phenotype data from 2019–2021 Planted at the experimental base of Jilin Agricultural University, such as the contents of protein and fat of soybeans. Through the GLM model and MLM model, four SNP sites (Gm09_39012959, Gm12_35492373, Gm16_9297124, and Gm20_24678362) that were significantly related to soybean fat content were associated for three consecutive years, and two SNP sites (Gm09_39012959 and Gm20_24678362) that were significantly related to soybean protein content were associated. By the annotation and enrichment of genes within the 100 Kb region of SNP loci flanking, two genes (*Glyma.09G158100* and Glyma.09G158200) related to soybean protein synthesis and one gene (*Glyma.12G180200*) related to lipid metabolism were selected. By the preliminary verification of expression levels of genes with qPCR, it is found that during the periods of R6 and R7 of the accumulation of soybean protein and fat, *Glyma.09G158100* and *Glyma.09G158200* are positive regulatory genes that promote protein synthesis and accumulation, while *Glyma.12G180200* is the negative regulatory gene that inhibits fat accumulation.

**Conclusions:**

These results lay the basis for further verifying the gene function and studying the molecular mechanisms regulating the accumulation of protein and fat in soybean seeds.

**Supplementary Information:**

The online version contains supplementary material available at 10.1186/s12864-023-09687-6.

## Introduction

Originating in China, soybeans were cultivated as early as 5000 years ago [1]. Currently, 65% of vegetable proteins and 31% of edible oils consumed by humans are derived from soybeans [2]. Studies show that relevant functional components of soybean proteins have a positive role in improving mammalian renal function, preventing cardiovascular diseases, and decreasing cholesterol levels [3]. After feeding adult dogs with soybean proteins for three weeks, Kontesis et al. found that the clearances of dogs' renal plasma flow, glomerular filtration rate, and renal albumin were all decreased [4]. Ohara et al. provided conglycinin to 60 adult volunteers with a lipid content of 1.7–3.39 mmol/L. After taking it for four weeks, these adults' blood lipid levels decreased by 25% [5]. Ascencio et al. found that soybean proteins could reduce the accumulation of cholesterol and triglycerides to prevent fatty liver [6]. Furthermore, soybean oil is rich in active ingredients, such as fatty acids, phospholipids, and tocopherols (vitamin E). Among them, fatty acids have significant anti-tumor effects [7]; as a health product, phospholipids are the source of ammonium polyphosphate in human bodies, playing an important role in regulating hepatic steatosis and cholesterol metabolism and lowering blood lipid levels [8]; vitamin E has promotional effects on human physiological functions and regulating endocrine and immune functions [9]. Therefore, improving the contents of soybean protein and fat is significant to satisfy the needs of food, pharmaceutical, and health industries.

As quantitative traits, soybean protein and fat are regulated by multiple genes and susceptible to environmental conditions [10]. Genome-wide association study (GWAS) is currently an ideal method for studying quantitative traits and mining related genes. In contrast with other mapping methods, GWAS has low requirements for populations. It only needs natural populations to perform association analysis on target traits instead of additional construction of dual or multi-parent colonies. Moreover, it has high marker density and positioning accuracy, greatly improving the efficiency and accuracy of quantitative trait loci mapping. In recent years, there have been a large number of studies using GWAS to carry out positioning experiments on soybean yields and quality traits [11]. Javaid et al. evaluated four yield-related traits in six environments by GWAS association analysis, identified six important SNPs, and determined 15 candidate genes through annotation and expression analysis [12]. Zhao et al. measured the contents of unsaturated fatty acids in 196 soybean germplasms for three consecutive years and conducted GWAS. A total of 147 SNP loci were associated with unsaturated fatty acid contents. Through annotation, 12 new genes that might regulate the synthesis of unsaturated fatty acids were selected [13]. Lu et al. used 278 different soybean germplasms to identify quantitative trait nucleotides (QTNs) of soluble sugar in soybeans based on GWAS. 17 genes affecting soluble sugar were identified among 115 genes on the flanks of 13 SNPs [14].

Although GWAS based on resequencing technology has visible advantages in marker density, due to the limitation of population sizes and sequencing costs, it is difficult to make good use of its advantages. In recent years, specific-length amplified fragment sequencing (SLAF-seq) has been applied in GWAS [15]. Compared to re-sequence's needing to smash genomes without distinction through ultrasounds and sequence and splice the broken fragments, SLAF-seq only needs to undergo systematic evaluation, select restriction endonucleases that can evenly cleave genomes, sequence the cleaved fragments one by one, and splice them according to the cleaved sites. SLAF-seq optimizes labeling efficiency on the basis of re-sequence and ensures the labeling density while reducing the difficulty and costs of sequencing [16, 17]. In summary, GWAS based on SLAF-seq is more suitable for the research targeting medium populations.

This study selected 292 different soybean germplasms from Northeast China to investigate the contents of protein and fat of soybeans for three consecutive years. Based on the SLAF-seq technology and combined with the phenotypic data, GWAS was used to annotate and screen 100 kb flanking genes of associated SNP loci. A total of three genes related to protein and fat syntheses were screened. According to the verification of qPCR expression, during the accumulation period of fat and protein in soybean seeds, the gene related to soybean fat hydrolysis (*Glyma.12G180200*) was significantly downregulated, while the genes related to soybean protein synthesis (*Glyma.09G158100* and *Glyma.09G158200*) were significantly upregulated. The research results have critical guiding significance for applying molecular-assisted breeding to improve the contents of soybean protein and fat.

## Materials and methods

### Experimental materials and cultivate management

Two hundred ninety-two soybean germplasm materials used in the experiment were provided by the Biotechnology Center of Jilin Agricultural University (Additional file 1: Table S1). Experiments were carried out in the experimental base of Jilin Agricultural University (43° 88'N, 12,535'E) in 2019, 2020, and 2021, respectively. Each variety was planted in four rows, with a row length of four meters, a row spacing of 0.65 m, and three replicates. The 292 cultivated soybeans were planted using a completely randomized design.

### Trait identification and statistical analysis

After the mature stage of soybeans was finished, selected the middle two rows from the four rows of each variety (to completely eliminate marginal effects), and randomly harvested 10 plants from the selected two rows. After threshing, whole soybeans were used as experimental materials. Each variety was measured three times. The average value was taken as the moisture content of the variety of soybeans [18]. The contents of protein and fat in soybean seeds after drying were measured with a NIRS DS2500 NIR mass spectrometer. Each variety was performed technical measurements three times. The data was analyzed through repetition and average values. The R programming language of version 4.1.3 (https://www.r-project.org/) was adopted to obtain descriptive statistics and construct frequency distribution histograms. The related data of seed protein and fat was performed ANOVA analysis and broad heritability calculation [19].

The estimated broad heritability ${h}^{2}$ is:

$${h}^{2}={\sigma }_{G}^{2}/\left({\sigma }_{G}^{2}+{\sigma }_{GE}^{2}/n+{\sigma }_{e}^{2}/nr\right)$$

For various environments:

$${h}^{2}={\sigma }_{G}^{2}/\left({\sigma }_{G}^{2}+{\sigma }_{e}^{2}\right)$$

${\sigma }_{G}^{2}$ denotes the genotypic variance; ${\sigma }_{GE}^{2}$ denotes the variance of the interaction between the genotype and the environment; ${\sigma }_{e}^{2}$ denotes the error variance; _n_ denotes the number of environments; _r_ denotes the repeated time in each environment.

### SNP Genotyping and quality control

When the terately compound leaves of soybeans grew up, the second leaves were used as materials. The genomic DNA of 292 soybean lines was extracted by the updated CTAB method [20]. The concentration and purity of DNA were detected using a NANODROP 2000 spectrophotometer (Thermo Scientific, Barrington, IL, USA). Qualified samples were used for SNP genotyping. The genomic DNA of 292 soybean natural populations of all lines was sequenced using the SLAF-seq technology. The obtained original data was filtered. The SLAF-seq sequencing method referred to Fei [21] and others. Glycine_max:Wm82.a4.v1 was used as the reference genome, and the sequencing reads were aligned with the reference genome using bwa [22]. Two methods, GATK [23] and samtools [24], were adopted to develop SNP. The intersection of the SNP markers obtained by the two methods was used as the final reliable SNP marker dataset. To guarantee the quality of the SNP, the SNP filtering criteria were: minor allele frequency: MAF > 0.05; locus integrity: INT > 0.5. A total of 641,542 collective SNP were obtained. After filtering, high-quality SNP variable sites obtained were performed functional annotation for subsequent GWAS analysis.

### Phylogenetic tree and population structure analysis

Based on the high-quality collective SNPs, this study used the software of MEGA X [25] to analyze. The distance matrix was generated through calculation. According to the neighbor-joining method, the p-distance distance was used to calculate models. Repeated 1000 times by bootstrap and constructed a phylogenetic tree.

Based on the SNPs population, the population structure of research materials was analyzed using the software of admixture [26]. For the study population, the preset number of subgroups (K value) was 1–6 to cluster. The clustering results were cross verified. The optimal number of subgroups was determined based on the value of the cross-validation error rate. 641,542 SNP markers were performed principal component analysis by the software of EIGENSOFT [27]. The genetic relationship between two individuals in the natural population was estimated by GCTA [28] software. The genetic relationship can be divided into A matrix and G matrix. The A matrix is generally calculated through phylogenetic relationship, and the G matrix is generally calculated through genetic markers (in this paper, it refers to SNP markers). By default, this study used the mean of the expected marker variance to adjust the expected marker variance, the G matrix [29].

### GWAS

GWAS was conducted based on 641,542 SNPs of minor allele frequency (MAF) > 5%. The population structure matrix (Q) and kinship matrix (K) were obtained through calculation. The Bonferroni threshold *P* < 1.0 × 10–3 (- log10 *P* < 3.0) was used to explain the significant correlation between SNP loci and traits. Two models, GLM [30] and MLM [31], were used in the analysis. In these two models, the Q and K matrices acted as fixed and random effects, respectively. SNP sites simultaneously detected in two models in three environments could be considered stable and consistent in this study [32].

### Screening and annotation of candidate genes

According to the physical locations of significant SNP markers obtained from screening in the reference genome Glycine_max:Wm82.a4.v1 and the range of 100kbp flanking regions at the peaks of significant SNPs, candidate genes related to target traits were predicted. Candidate genes were conducted GO ( http://geneontology.org/) and KEGG (https://www.kegg.jp/) functional annotations. The annotation results were performed enrichment analysis to determine the existence of the function related to the content of soybean protein or fat in the gene.

### qPCR

Genes related to protein and fat were conducted expression detection with the technology of qRT-PCR. The sampling and detection periods for the expression of protein and fat-related genes were R6 (On the top 4 fully spread compound leaves of the main stem of soybeans, one of the pods at any node fills the pod cavity) and R7 (There is a pod on the main stem of the soybean that reaches a mature color), respectively. This study selected five varieties with high-phenotypic values and five varieties with low-phenotypic values for the two traits as experimental materials (Additional file 2: Table S2). The selected soybean varieties were cultivated in plant incubators under a constant temperature of 26 ℃ with 18 h of light and 6 h of darkness. Testing materials were collected on-site in 2022. Samples harvested during R6 (soybean seed filling period) and R7 (soybean maturity period) were used to extract total RNA using RNAiso Plus (EasyPure® Plant RNA Kit, Beijing, China) and reversely transcribed cDNA by the All-in-One™ First t-Strand cDNA Synthesis Kit (GeneCopoeia Inc., United States). IDT-dna (https://sg.idtdna.com/pages) was used to design qPCR primers (Additional file 3: Table S3). Agilent Stratagene Mx3000P (Palo Alto, CA, USA) was adopted for qPCR detection. The method of 2^−ΔΔCt^ [33] was used to calculate expressions. (Additional file 4: Table S4). One-way ANOVA used for statistical analysis of qPCR experiments. Histograms were plotted by Graphpad Prism 9.5.0 (https://www.graphpad-prism.cn/).

## Results

### Phenotypic data analysis of the contents of soybean protein and fat

**Fig. 1:**
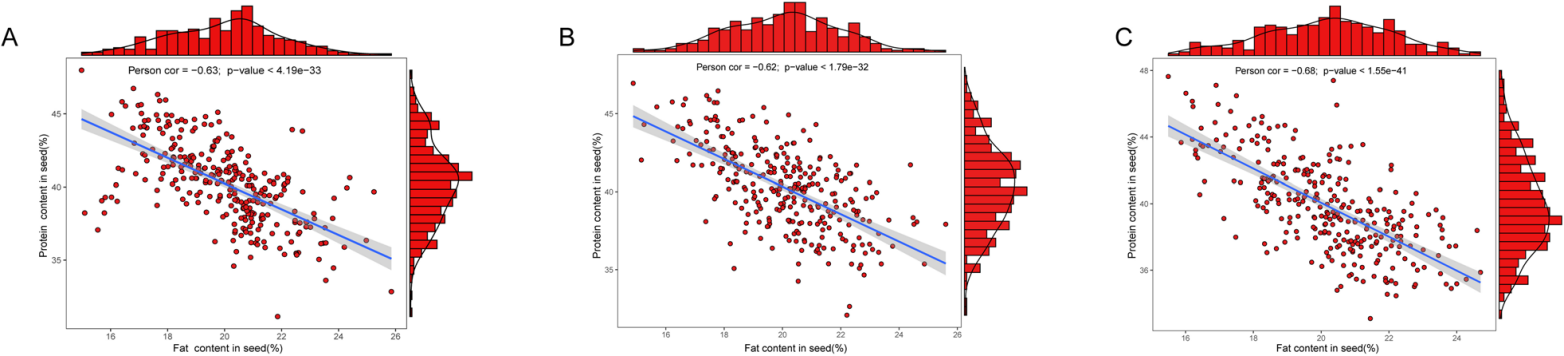
Distribution and correlation analysis of the contents of soybean protein and fat in different years. **A**: 2019; **B**: 2020; **C**: 2021

This experiment used 292 soybean materials from different regions. They were cultivated and conducted post-harvest quality detection in 2019, 2020, and 2021, respectively. The contents of protein and fat of soybeans were reorganized (Additional file 5: Table S5). The analysis results of three-year phenotypic data show that the protein and fat contents in the natural population are normally distributed, and the two traits are negatively correlated (Fig. 1). The results of variance analysis show that under 3-year planting conditions, the two traits had extensive mutations in the population, and there were significant differences (*p* < 0.01). The estimated values of broad heritability (H^2^) were both high (Table 1), indicating that the genetic effect had significant impacts on protein and fat contents.

**Table 1:** Descriptive statistical results of the traits of soybean fat and protein contents under the three-year environment

Traits	Years	Mean	^±^SD	Median	Min	Max	Range	h^2^	F Values from ANOVA		
									Line	Env	Line × Env
Oil	2019	19.99	1.98	20.17	14.98	25.85	10.87	0.82	2.17^***^	0.01^ ns^	0.95^ ns^
	2020	20.07	1.89	20.17	14.88	25.60	10.72				
	2021	20.17	1.88	20.27	15.49	24.70	9.21				
Protein	2019	40.23	2.79	40.34	31.13	47.99	16.86	0.73	3.48^***^	0.05^*^	0.57^ ns^
	2020	40.27	2.68	40.20	32.08	46.95	14.87				
	2021	39.89	2.81	39.57	33.09	47.64	14.55				

^***, **, *^ and ^ns^ represent the significant levels of *P*<0.001, *P*<0.01, *P*<0.05 and ns: No significance respectively

### Genotyping and population structure

**Fig. 2:**
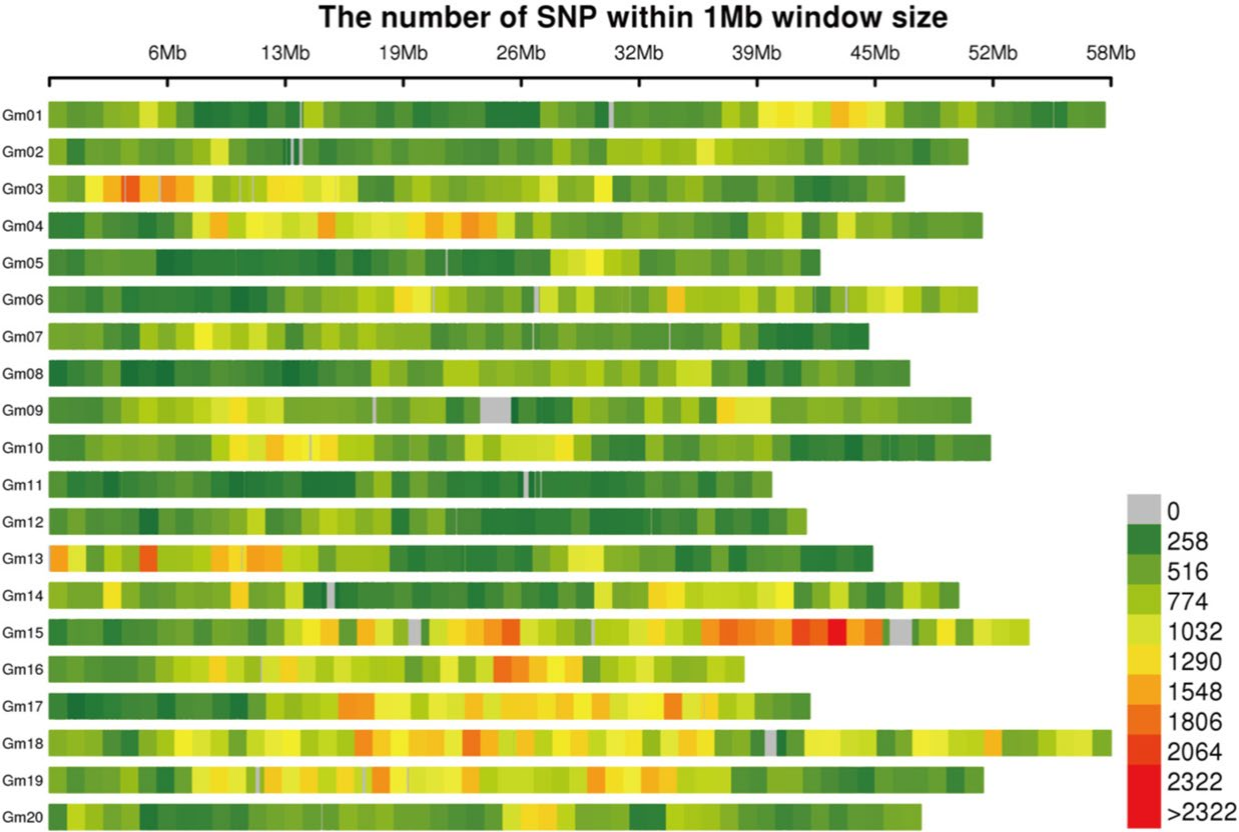
The horizontal ordinate is the length of chromosomes. Each band represents a chromosome. The genome is divided according to the size of 1 Mb. The more SNP markers, the darker the color. The less SNP markers, and the lighter the color. The darker area in the graph is the area where the distribution of SNP markers is concentrated

The SLAF-seq technology was used for the development of molecular markers of 292 materials in this study. Glycine_max:Wm82.a4.v1 was selected as the reference genome for electronic enzyme digestion prediction, and it was determined to use RsaI + HaeIII for enzyme digestion. A total of 1,485.09 Mb reads were obtained, with an average content of Q30 of 93.88% and an average content of GC of 39.96%. Through bioinformatics analysis, a total of 473,597 SLAF markers were obtained, including 164,737 polymorphic SLAF markers and 641,542 collective SNP markers (Fig. 2).

**Fig. 3:**
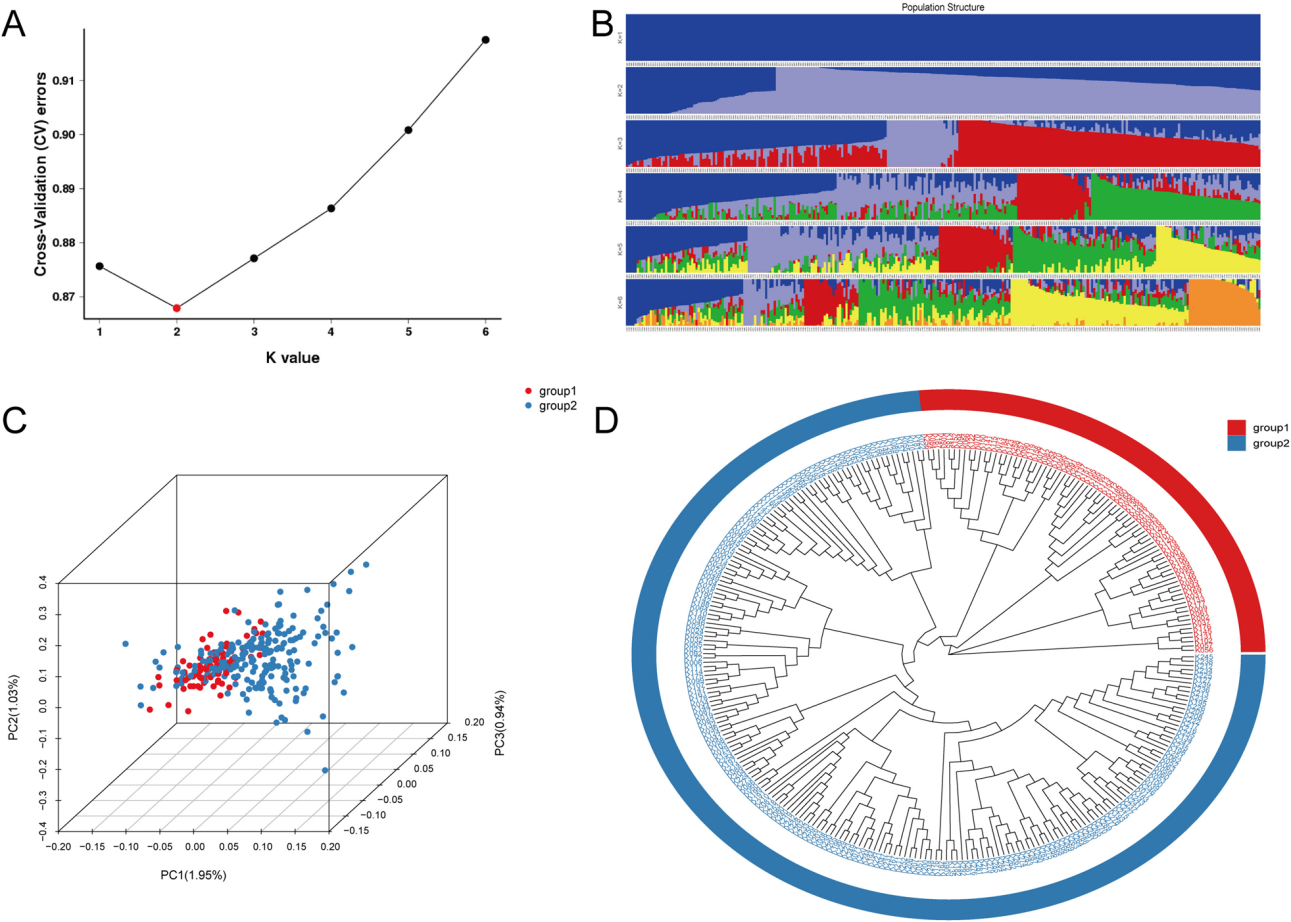
Population structure of 292 soybean genotypes. **A** Based on the population, the number of subgroups (K value) is preset to 1–6 for clustering and cross-validation to further determine the optimal number of subgroups. Cluster results when the value of K ranges from 1–6 and the cross-validation error rate corresponding to each K; **B** Cluster analysis. Different colors represent corresponding groups. **C** Soybean lines from three geographical locations are distinguished by PCI, PC2, and PC3. **D** The phylogenetic tree of 292 soybean materials

Two hundred ninety-two genotypic materials were divided into two subpopulations based on the results of the K value analyzed by the admixture software. The phylogenetic tree shows that the soybean materials used in this study derived from three major branches, further proving that 292 soybean materials had the same ancestor. However, subsequent evolution was completed in different subgroups (Fig. 3A, B). The cluster analysis was conducted based on the degree of SNP difference by principal component analysis (PCA). The analysis results show that the 292 materials could be divided into different subgroups based on more overlapping areas (Fig. 3C), with PC1, PC2, and PC3 accounting for 1.95%, 1.03%, and 0.94%, respectively. Perhaps we can understand that 292 soybean materials are divided into two subgroups, representing a mixture of three ancestral populations (Fig. 3D).

### GWAS Analysis on the contents of soybean protein and fat

In this study, the contents of soybean protein and fat were mostly influenced by genetic factors. To further determine significant SNPs associated with target traits, two GWAS models (GLM and MLM) were applied to analyze high-quality SNPs of 292 soybean germplasms used in the experiment under a critical threshold of − log10 (*P*) < 3.0 to obtain accurate and stable genetic loci.

**Fig. 4:**
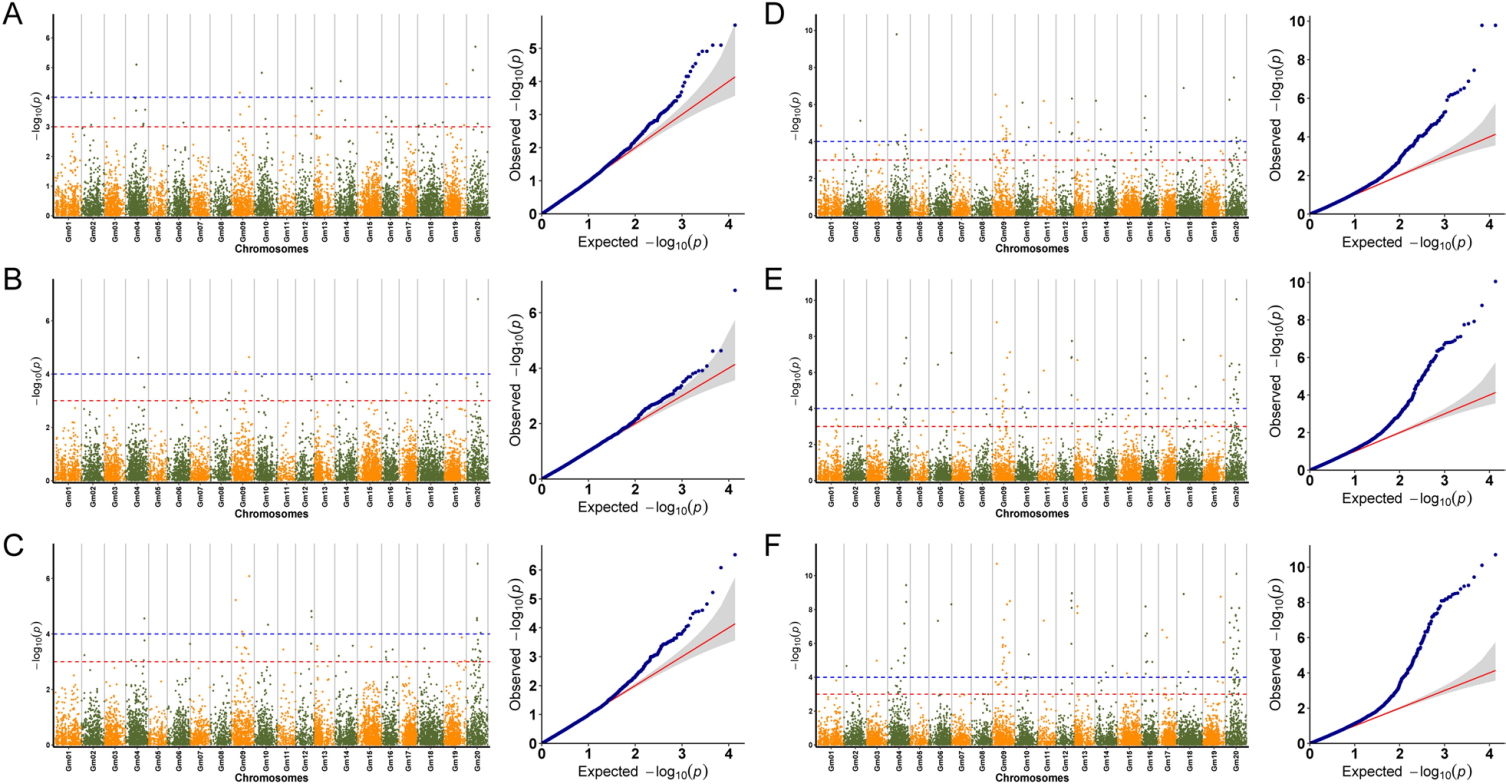
Based on the phenotypic data of soybean fat content from 2019, 2020, and 2021, by the analysis of the two models, visualize Manhattan Plot and Q-Q graph. **A B C** are the Manhattan Plot and Q-Q graph of the soybean fat of the GLM model in 2019, 2020, and 2021; **D E F** are the Manhattan Plot and Q-Q graph of the soybean fat of the MLM model in 2019, 2020, and 2021

Thirty-nine, 25, and 62 SNP loci related to soybean fat content were detected by the GLM model in 2019, 2020, and 2021, respectively (Fig. 4A, B, C). These SNP loci were distributed on 17 chromosomes. No related loci were found on Chr.01, Chr.05, and Chr.07. A total of 106 SNPs were detected in three-year environments (2019, 2020, 2021). By comparing the results, it was found that 90 of the 106 SNPs were sensitive to environments under different year conditions, with 12 SNPs detected under two-year conditions and four SNPs detected under three-year conditions (Fig. 6A). 124, 125, and 158 SNP sites related to soybean fat content were detected by the MLM model in three years, and a total of 232 SNPs were detected, of which 73 were detected in two-year environments, and 51 were detected in three-year environments (Figs. 4D, E, F, and 6B).

Through the comparison of the results of different GWAS models, we found that under three-year environments, both models were located at four consistent SNP sites (Gm09_39012959, Gm12_35492373, Gm16_9297124, and Gm20_24678362) (Fig. 6C). We can consider that these four SNPs are genetically stable and closely related to soybean fat content.

**Fig. 5:**
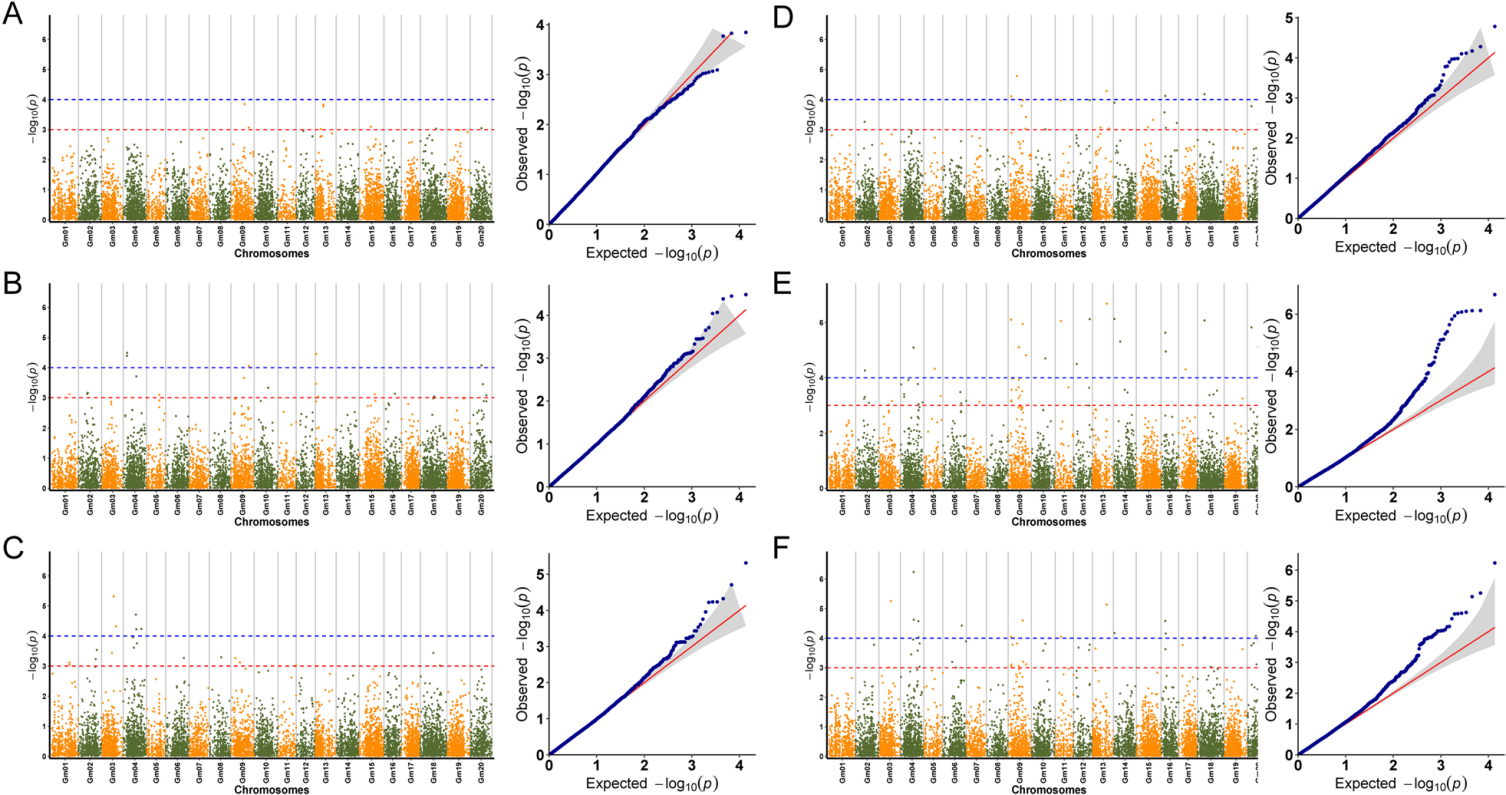
Based on the phenotypic data of soybean protein content from 2019, 2020, and 2021, by the analysis of the two models, visualize Manhattan Plot and Q-Q graph. **A B C**) are the Manhattan Plot and Q-Q graph of the soybean protein of the GLM model in 2019, 2020, and 2021; **D E F** are the Manhattan Plot and Q-Q graph of the soybean protein of the MLM model in 2019, 2020, and 2021

**Fig. 6:**
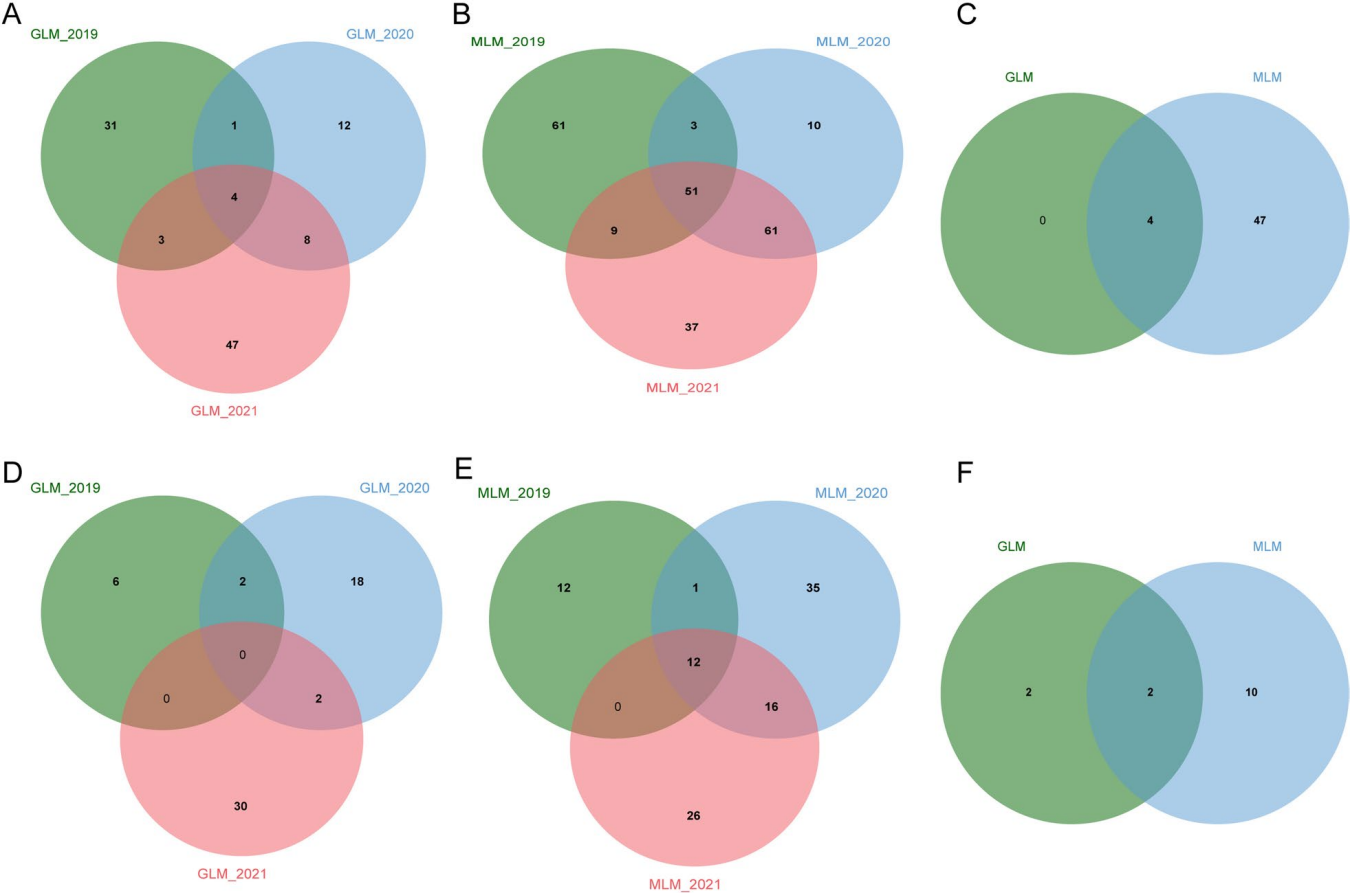
Statistics of significant SNP loci related to fat and protein in GLM and MLM models from 2019 to 2021. **A** The number of significant SNP loci obtained by the GLM model for fat in the years 2019, 2020, and 2021, respectively; **B** The number of significant SNP loci obtained by the MLM model for fat in the years 2019, 2020, and 2021, respectively; **C** The number of significant SNP loci existing in both GLM and MLM models of fat under three-year conditions; **D** The number of significant SNP loci obtained by the GLM model for protein in the years 2019, 2020, and 2021, respectively; **E** The number of significant SNP loci obtained by the MLM model for protein in the years 2019, 2020, and 2021, respectively; **F** The number of significant SNP loci existing in both GLM and MLM models of protein in the years 2019, 2020, and 2021, respectively

By analyzing soybean protein content, it is found that 8, 22, and 32 SNP loci related to soybean protein content were detected by the GLM model in 2019, 2020, and 2021, respectively (Fig. 5A, B, C). A total of 58 SNPs were detected in the past three years. Four SNPs were detected in two years, which were distributed on Chr.01, Chr.04, Chr.09, and Chr.20, respectively (Fig. 6D). 25, 64, and 54 SNP loci were detected by the MLM model in three years, respectively, of which 17 SNPs were detected in two years, and 12 SNPs were detected in three years (Figs. 5D, E, F, and 6B).

By comparing the results of the two models, it is shown that the two models have simultaneously located two identical SNP loci (Gm09_39012959, Gm20_24678362) under three-year environmental conditions (Fig. 6F).

### Candidate gene prediction and qRT-PCR analysis

**Fig. 7:**
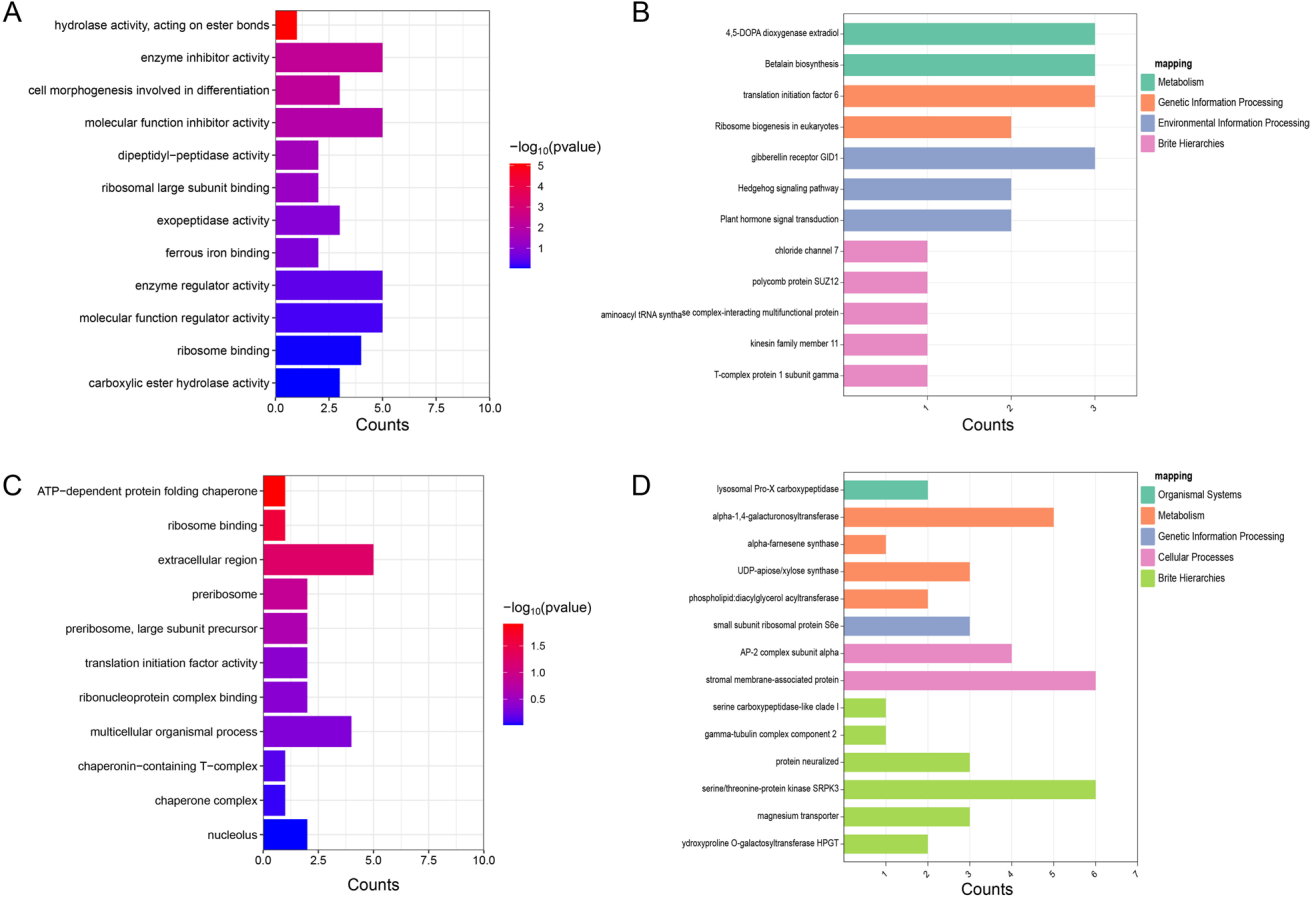
Enrichment analysis of candidate genes related to the contents of fat and protein. **A** GO enrichment analysis of candidate genes related to soybean fat content; **B** KEGG enrichment analysis of candidate genes related to soybean fat content; **C** GO enrichment analysis of candidate genes related to soybean protein content; **D** KEGG enrichment analysis of candidate genes related to soybean protein content [34–36]

Go and KEGG annotations were carried out to the 100 Kb flanking genes of consistent SNP sites related to fat content, Gm09_39012959, Gm12_35492373, Gm16_9297124, and Gm20_24678362. The results show that genes mainly concentrate on pathways related to fat metabolism and protein synthesis. A GDSC lipase family-gene (*Glyma.12G180200*) related to fat metabolism is annotated in the 100 Kb flank of Gm12_35492373. The KEGG annotation of the gene is on the glycerol metabolism pathway. The results of the 100 kb flanking annotation of consistent SNPs related to protein content (Gm09_39012959 and Gm20_24678362) show that gene functions mainly concentrate in protein synthesis, hormone regulation, and kinase activity. In the 100 Kb flank of Gm09_39012959, the two genes of *Glyma.09G158100* and *Glyma.09G158200* are annotated as a translation initiation factor and peptide chain folding, respectively, which are related to protein synthesis and accumulation (Fig. 7).

Therefore, we consider that *Glyma.12G180200*, *Glyma.09G158100*, and *Glyma.09G158200* (Additional file 6: Table S6) are candidate genes.

**Fig. 8:**
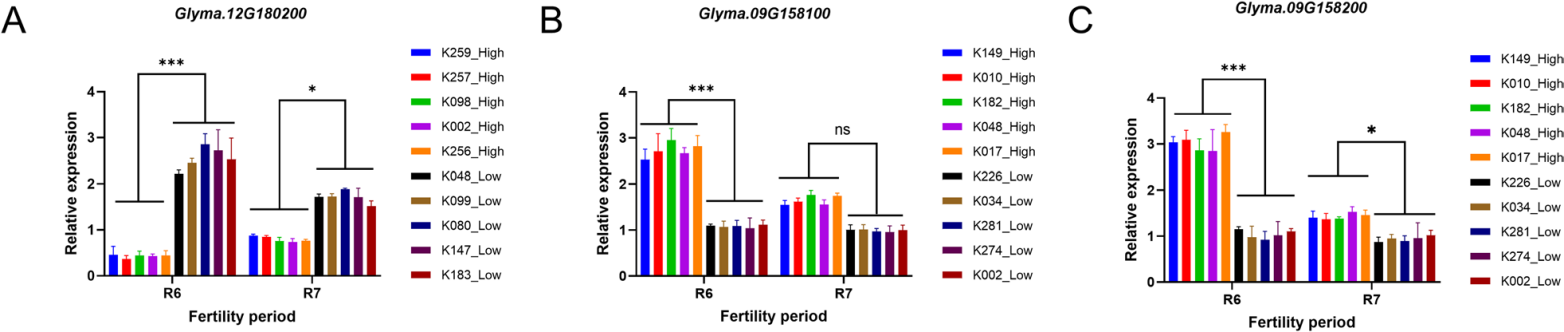
Relative expressions of three candidate genes in the contents of soybean fat and protein are analyzed by qRT-PCR. The first five materials in the figure represent the average gene expressions of high-fat and high-protein materials at R6 (soybean seed filling period) and R7(soybean maturity period), while the latter five materials represent the average gene expressions of low-fat and low-protein materials at R6 (soybean seed filling period) and R7(soybean maturity period) (the results in the above figure use soybean seeds as experimental materials).***, * and ns represent the significant levels of *P* < 0.001, *P* < 0.05 and ns: No significance respectively. **A** Fat-related genes validated for expression in some population materials; **B**, **C** Protein-related genes validated for expression in some population materials

The qPCR expression analysis of three candidate genes shows that compared with the low-fat germplasm, the expression of *Glyma.12G180200* in the high-fat germplasm R6 (soybean seed filling period) is significantly downregulated, which is lower than that in R7(soybean maturity period). In the R6 (soybean seed filling period) of efficient fat accumulation, the expression of *Glyma.12G180200* is inhibited, while in the low-fat germplasm, the expression is significantly upregulated. The expression patterns of *Glyma.09G158100* and *Glyma.09G158200* are similar, with the highest expression level at the R6 (soybean seed filling period), which is significantly different from the control material, while at the R7(soybean maturity period), the expression level is significantly reduced but still significantly different from the control material (Fig. 8).

## Discussion

Based on the SLAF-seq sequencing technology [37], this study conducted GWAS on the contents of protein and fat in a natural population containing 292 soybean germplasms, reorganized the phenotypic data from three points in three years, and associated four consistent SNP sites related to soybean fat content and two related to soybean protein content, which were located on chromosomes 9, 12, 16, and 20, respectively. Among them, Gm09_39012959 and Gm20_24678362 are pleiotropy SNP sites simultaneously associated with contents of both protein and fat of soybeans [38]. Comparing the above results with previous studies, In this study, the SNP site Gm09_39012959 related to soybean protein and fat was located, and the QTL (592,513–4,266,665) significantly related to soybean fat content was located by Msnsur et al. [39] using F2 isolation population. Diers et al. [40] used RFLP molecular markers to locate QTL in the F2 population and identified a major QTL (24,177,772–34,074,375) that regulated soybean fat content. Sebolt [41] also located a QTL related to fat content in the exact physical location, and both of these results physically overlapped with the SNP site Gm 20_24678362 located in this study. In addition, the positioning results of this study in different years are primarily similar to those of previous studies, which all show that the positioning results of this study are very reliable. Although previous studies have not located a multi-effect site that overlaps with Gm 09/39012959 and Gm 20_24678362, a multieffect site for soybean fat and protein content is accurate. Hwang et al. [42] located quantitative trait loci (QTLS) related to soybean protein and fat content based on 298 soybean varieties and found seven markers related to protein and fat content on chromosomes 8, 9, and 20. The presence of pleiotropic sites further indicates that the metabolic pathways of protein and fat in soybeans are related. In addition, we identified two new SNPS, Gm 12_35492373 (35,492,373) and Gm 16_9297124 (9,297,124). In recent years, there have been new reports that many reliable genetic loci related to protein and fat content have been detected in different populations and regions. This suggests that genetic loci may be controlled by specific alleles, perhaps only in specific materials. The soybean germplasm resources used in this study come from different regions, have a comprehensive genetic background, and were identified in different years. Therefore, the discovery of these two new loci may be due to the use of new populations and growth conditions. Therefore, we used diverse soybean germplasm to locate results consistent with previous generations and found new reliable genetic loci related to soybean fat and protein.

The accumulation of fat and protein in plant seeds is closely related to their anabolism [43]. Triacylglycerol is the major preservative lipid of plants, which synthesizes fat with acetic acids through ester bonds. The GDSC lipase family has hydrolytic activity, which can hydrolyze ester bonds and cleave esters into alcohols and acids [44]. We screened a gene of GDSC lipase family, *Glyma.12G180200*, from a consistent SNP locus (Gm12_35492373) closely related to fat content. The GO annotation indicates that *Glyma.12G180200* has hydrolytic activity, and the KEGG annotation represents that *Glyma.12G180200* participates in the glycerol metabolism pathway. By verifying the qPCR expression of this gene, the results show that during the R6 period, the expression levels of soybean germplasms with low-fat contents are significantly higher than that of germplasms with high-fat contents. Generally, *Glyma.12G180200*, as a member of the GDSC lipase family, may play a hydrolase role in the glycerol metabolism pathway, hydrolyzing fats into alcohols and acids and negatively regulating the accumulation of fats [45].

Proteins stored in soybeans mainly consist of globulin and β-conglycinin [46]. The pathways involved chiefly include protein synthesis and processing, protein sorting and storage, and protein accumulation [47]. In the process of protein synthesis, the 40 s small subunit combines with translation initiation factors, and then translation initiation factors bind to the mRNA template. With the assistance of translation initiation factors and GTP, Met-tRNA enters the small subunit, and the anticodon on tRNA pairs with the start codon on mRNA. Finally, the small subunit composed of tRNA, mRNA, and translation initiation factors combines with the large subunit. Along with GTP hydrolysis, translation initiation factors are released [48]. Peptides synthesized from ribosomes are then folded through endoplasmic reticulum to become mature proteins of three-dimensional structure [49]. We screened two genes related to protein synthesis, *Glyma.09G158100* and *Glyma.09G158200*, from a consistent SNP locus closely associated with protein content (Gm09_39012959). The GO annotation of *Glyma.09G158100* is a transcription initiation factor, a member of the transcription initiation factor family of eIF6. The GO annotation of *Glyma.09G158200* is endoplasmic reticulum folding, which is an important part of endowing peptide chains with tertiary structures. The analysis of the expression levels of *Glyma.09G158100* and *Glyma.09G158200* shows that the expressions of both genes are significantly upregulated during the period of R6, indicating that these two genes may play a crucial role in protein synthesis and accumulation.

## Conclusions

Four SNP sites related to fat in soybean seeds and two SNP sites related to protein content in soybean seeds that have been stable for three consecutive years were associated in this study by GWAS based on the GLM and MLM models. Through annotation mining and expression verification, the gene *Glyma.12G180200* of the GDSC lipase family, which played a negative regulatory role in fat accumulation, and *Glyma.09G158100* and *Glyma.09G158200*, which played a positive regulatory role in protein synthesis, were identified. These results lay the ground for the molecular mechanism, genetic structure, and molecular marker-assisted breeding of the contents of soybean fat and protein [50].

### Supplementary Information

**Additional file 1: Table S1. **Geographical distribution of 292 soybean germplasm resources.

**Additional file 2: Table S2. **This study selected five varieties with high-phenotypic values and five varieties with low-phenotypic values for the two traits as experimental materials.

**Additional file 3: Table S3. **List of primers used for the qPCR assay of the key structural genes involved in protein and fat traits.

**Additional file 4: Table S4.**MIQE Guidelines:qPCR details.

**Additional file 5: Table S5. **Three-year phenotypic data on fat and protein from 292 soybean germplasm resources.

**Additional file 6: Table S6. **Information on candidate genes related to fat and protein content of soybean.

## Data Availability

The sequencing data from this study has been uploaded to NCBI (PRJNA940512) and all remaining data generated or analysed are included in this published article and its supplementary information file.
