# Supplementary material for: Genome-wide association study and high-quality gene mining related to soybean protein and fat
Source: BMC Genomics. 2023 Oct 7;24:596. doi: 10.1186/s12864-023-09687-6 (PMC10559447; doi:10.1186/s12864-023-09687-6)
Supplement: Supplementary file 1 — Additional file 1: Table S1. Geographical distribution of 292 soybean germplasm resources. [file 12864_2023_9687_MOESM1_ESM.docx]

**Table S1.** Geographical distribution of 292 soybean germplasm resources.

| Variety number | Regional origin |
| --- | --- |
| K001 | Jilin Province |
| K002 | Jilin Province |
| K003 | Jilin Province |
| K004 | Jilin Province |
| K005 | Jilin Province |
| K006 | Jilin Province |
| K007 | Jilin Province |
| K008 | Jilin Province |
| K009 | Jilin Province |
| K010 | Jilin Province |
| K011 | Jilin Province |
| K012 | Jilin Province |
| K013 | Hebei Province |
| K014 | Hebei Province |
| K015 | Hebei Province |
| K016 | Hebei Province |
| K017 | Hebei Province |
| K018 | Hebei Province |
| K019 | Hebei Province |
| K020 | Hebei Province |
| K021 | Hebei Province |
| K022 | Hebei Province |
| K023 | Hebei Province |
| K024 | Hebei Province |
| K025 | Hebei Province |
| K026 | Heilongjiang Province |
| K027 | Heilongjiang Province |
| K028 | Heilongjiang Province |
| K029 | Heilongjiang Province |
| K030 | Heilongjiang Province |
| K031 | Heilongjiang Province |
| K032 | Heilongjiang Province |
| K033 | Heilongjiang Province |
| K034 | Heilongjiang Province |
| K035 | Heilongjiang Province |
| K036 | Heilongjiang Province |
| K037 | Heilongjiang Province |
| K038 | Heilongjiang Province |
| K039 | Heilongjiang Province |
| K040 | Heilongjiang Province |
| K041 | Heilongjiang Province |
| K042 | Heilongjiang Province |
| K043 | Heilongjiang Province |
| K044 | Heilongjiang Province |
| K045 | Heilongjiang Province |
| K046 | Heilongjiang Province |
| K047 | Heilongjiang Province |
| K048 | Sichuan Province |
| K049 | Sichuan Province |
| K050 | Sichuan Province |
| K051 | Sichuan Province |
| K052 | Sichuan Province |
| K053 | Sichuan Province |
| K054 | Sichuan Province |
| K055 | Inner Mongolia |
| K056 | Inner Mongolia |
| K057 | Inner Mongolia |
| K058 | Inner Mongolia |
| K059 | Inner Mongolia |
| K060 | Inner Mongolia |
| K061 | Inner Mongolia |
| K062 | Inner Mongolia |
| K063 | Inner Mongolia |
| K064 | Inner Mongolia |
| K065 | Inner Mongolia |
| K066 | Inner Mongolia |
| K067 | Inner Mongolia |
| K068 | Inner Mongolia |
| K069 | Italy |
| K070 | Italy |
| K071 | Italy |
| K072 | Italy |
| K073 | Italy |
| K074 | Italy |
| K075 | Italy |
| K076 | Italy |
| K077 | Italy |
| K078 | Italy |
| K079 | Italy |
| K080 | Japan |
| K081 | Japan |
| K082 | Japan |
| K083 | Japan |
| K084 | Japan |
| K085 | Japan |
| K086 | Japan |
| K087 | United States |
| K088 | United States |
| K089 | United States |
| K090 | United States |
| K091 | United States |
| K092 | United States |
| K093 | United States |
| K094 | United States |
| K095 | United States |
| K096 | United States |
| K097 | United States |
| K098 | Canada |
| K099 | Canada |
| K100 | Canada |
| K101 | Canada |
| K102 | Canada |
| K103 | Canada |
| K104 | Canada |
| K105 | Canada |
| K106 | Canada |
| K107 | Canada |
| K108 | Canada |
| K109 | Canada |
| K110 | Canada |
| K111 | Canada |
| K112 | Germany |
| K113 | Germany |
| K114 | Germany |
| K115 | Germany |
| K116 | Germany |
| K117 | Germany |
| K118 | Germany |
| K119 | Henan Province |
| K120 | Henan Province |
| K121 | Henan Province |
| K122 | Henan Province |
| K123 | Henan Province |
| K124 | Henan Province |
| K125 | Shandong Province |
| K126 | Shandong Province |
| K127 | Shandong Province |
| K128 | Shandong Province |
| K129 | Shandong Province |
| K130 | Shandong Province |
| K131 | Shandong Province |
| K132 | Shandong Province |
| K133 | Shandong Province |
| K134 | Shandong Province |
| K135 | Shanxi Province |
| K136 | Shanxi Province |
| K137 | Shanxi Province |
| K138 | Shanxi Province |
| K139 | Shanxi Province |
| K140 | Shanxi Province |
| K141 | Shanxi Province |
| K142 | Shanxi Province |
| K143 | Hebei Province |
| K144 | Hebei Province |
| K145 | Hebei Province |
| K146 | Hebei Province |
| K147 | Hebei Province |
| K148 | Shaanxi Province |
| K149 | Shaanxi Province |
| K150 | Shaanxi Province |
| K151 | Shaanxi Province |
| K152 | Shaanxi Province |
| K153 | Jiangsu Province |
| K154 | Jiangsu Province |
| K155 | Jiangsu Province |
| K156 | Jiangsu Province |
| K157 | Jiangsu Province |
| K158 | Jiangsu Province |
| K159 | Jiangsu Province |
| K160 | Jiangsu Province |
| K161 | Zhejiang Province |
| K162 | Zhejiang Province |
| K163 | Zhejiang Province |
| K164 | Zhejiang Province |
| K165 | Zhejiang Province |
| K166 | Liaoning Province |
| K167 | Liaoning Province |
| K168 | Liaoning Province |
| K169 | Liaoning Province |
| K170 | Liaoning Province |
| K171 | Liaoning Province |
| K172 | Hunan Province |
| K173 | Hunan Province |
| K174 | Hunan Province |
| K175 | Hunan Province |
| K176 | Hunan Province |
| K177 | Hunan Province |
| K178 | Hunan Province |
| K179 | Hubei Province |
| K180 | Hubei Province |
| K181 | Hubei Province |
| K182 | Hubei Province |
| K183 | Hubei Province |
| K184 | Hubei Province |
| K185 | Yunnan Province |
| K186 | Yunnan Province |
| K187 | Yunnan Province |
| K188 | Yunnan Province |
| K189 | Yunnan Province |
| K190 | Yunnan Province |
| K191 | Yunnan Province |
| K192 | Liaoning Province |
| K193 | Liaoning Province |
| K194 | Liaoning Province |
| K195 | Liaoning Province |
| K196 | Liaoning Province |
| K197 | Liaoning Province |
| K198 | Liaoning Province |
| K199 | Korea |
| K200 | Korea |
| K201 | Korea |
| K202 | Korea |
| K203 | Korea |
| K204 | Korea |
| K205 | Korea |
| K206 | Korea |
| K207 | Korea |
| K208 | Korea |
| K209 | Korea |
| K210 | Korea |
| K211 | Liaoning Province |
| K212 | Liaoning Province |
| K213 | Liaoning Province |
| K214 | Liaoning Province |
| K215 | Liaoning Province |
| K216 | Liaoning Province |
| K217 | Liaoning Province |
| K218 | Henan Province |
| K219 | Henan Province |
| K220 | Henan Province |
| K221 | Henan Province |
| K222 | Henan Province |
| K223 | Henan Province |
| K224 | Henan Province |
| K225 | Henan Province |
| K226 | Henan Province |
| K227 | Henan Province |
| K228 | Henan Province |
| K229 | Henan Province |
| K230 | Henan Province |
| K231 | Henan Province |
| K232 | Henan Province |
| K233 | Henan Province |
| K234 | Heilongjiang Province |
| K235 | Heilongjiang Province |
| K236 | Heilongjiang Province |
| K237 | Heilongjiang Province |
| K238 | Heilongjiang Province |
| K239 | Heilongjiang Province |
| K240 | Heilongjiang Province |
| K241 | Heilongjiang Province |
| K242 | Gansu Province |
| K243 | Gansu Province |
| K244 | Gansu Province |
| K245 | Gansu Province |
| K246 | Gansu Province |
| K247 | Gansu Province |
| K248 | Gansu Province |
| K249 | Gansu Province |
| K250 | Gansu Province |
| K251 | Gansu Province |
| K252 | Gansu Province |
| K253 | Gansu Province |
| K254 | Gansu Province |
| K255 | Gansu Province |
| K256 | Gansu Province |
| K257 | Gansu Province |
| K258 | Gansu Province |
| K259 | Gansu Province |
| K260 | Gansu Province |
| K261 | Jilin Province |
| K262 | Jilin Province |
| K263 | Jilin Province |
| K264 | Jilin Province |
| K265 | Jilin Province |
| K266 | Jilin Province |
| K267 | Jilin Province |
| K268 | Jilin Province |
| K269 | Jilin Province |
| K270 | Jilin Province |
| K271 | Jilin Province |
| K272 | Jilin Province |
| K273 | Jilin Province |
| K274 | Jilin Province |
| K275 | Jilin Province |
| K276 | Jilin Province |
| K277 | Jilin Province |
| K278 | Jilin Province |
| K279 | Jilin Province |
| K280 | Jilin Province |
| K281 | Jilin Province |
| K282 | Jilin Province |
| K283 | Jilin Province |
| K284 | Jilin Province |
| K285 | Jilin Province |
| K286 | Jilin Province |
| K287 | Jilin Province |
| K288 | Jilin Province |
| K289 | Jilin Province |
| K290 | Jilin Province |
| K291 | Jilin Province |
| K292 | Jilin Province |
