# Supplementary material for: Genome-wide association study and high-quality gene mining related to soybean protein and fat
Source: BMC Genomics. 2023 Oct 7;24:596. doi: 10.1186/s12864-023-09687-6 (PMC10559447; doi:10.1186/s12864-023-09687-6)
Supplement: Supplementary file 2 — Additional file 2: Table S2. This study selected five varieties with high-phenotypic values and five varieties with low-phenotypic values for the two traits as experimental materials. [file 12864_2023_9687_MOESM2_ESM.docx]

**Table S2.** This study selected five varieties with high-phenotypic values and five varieties with low-phenotypic values for the two traits as experimental materials.

| Line | Fat_content  (%) | Protein_content(%) | Line | Fat_content  (%) | Protein_content(%) |
| --- | --- | --- | --- | --- | --- |
| K259_High | 24.39 | 36.69 | K149_High | 17.93 | 45.95 |
| K257_High | 24.97 | 36.35 | K010_High | 15.47 | 46.33 |
| K098_High | 25.24 | 35.3 | K182_High | 16.8 | 46.74 |
| K002_High | 25.85 | 32.84 | K048_High | 14.98 | 44.37 |
| K256_High | 24.29 | 36.83 | K017_High | 17.72 | 45.9 |
| K048_Low | 14.98 | 44.37 | K226_Low | 25.86 | 31.13 |
| K099_Low | 15.09 | 46.18 | K034_Low | 22.32 | 34.59 |
| K080_Low | 15.55 | 41.9 | K281_Low | 24.55 | 33.61 |
| K147_Low | 15.69 | 41.1 | K274_Low | 23.82 | 34.46 |
| K183_Low | 15.78 | 42.71 | K002_Low | 25.85 | 32.84 |
