# Supplementary material for: Genome-wide association study and high-quality gene mining related to soybean protein and fat
Source: BMC Genomics. 2023 Oct 7;24:596. doi: 10.1186/s12864-023-09687-6 (PMC10559447; doi:10.1186/s12864-023-09687-6)
Supplement: Supplementary file 3 — Additional file 3: Table S3. List of primers used for the qPCR assay of the key structural genes involved in protein and fat traits. [file 12864_2023_9687_MOESM3_ESM.docx]

**Table S3.**List of primers used for the qPCR assay of the key structural genes involved in protein and fat traits.

| Name of Genes | Primer Sequences | Use |
| --- | --- | --- |
| *Glyma.12G180200* S  *Glyma.12G180200* AS | TCCTTGCTTCTGGTTGTAGTG  CTAAACACCACATCTCAGAGCT | qPCR assay |
| *Glyma.09G158100* S  *Glyma.09G158100* AS | CAGGGAGACTGAAGAGATGAT TG  TAATAGCGGCTGGAATGACAG | qPCR assay |
| *Glyma.09G158200* S  *Glyma.09G158200* AS | CTACACTCTTCCGCCGATTATT  GAAGAAGTCGGAGATGGAACG | qPCR assay |
| ActinII1S  ActinII1AS | GCTGTTCTTTCACTTTATGCAAG  CGCTCGGCTGAGGTGGTGAAGGA | Reference gene |
