# Supplementary material for: Genome-wide association study and high-quality gene mining related to soybean protein and fat
Source: BMC Genomics. 2023 Oct 7;24:596. doi: 10.1186/s12864-023-09687-6 (PMC10559447; doi:10.1186/s12864-023-09687-6)
Supplement: Supplementary file 4 — Additional file 4: Table S4.MIQE Guidelines:qPCR details. [file 12864_2023_9687_MOESM4_ESM.docx]

**Table S4.** MIQE Guidelines:qPCR details

|  | Item to check | Response |
| --- | --- | --- |
| Experimental design | Definition of experimental and control groups | Only the experimental group |
|  | Number within each group | 5 |
| Sample | Description | We selected 5 soybean plants with high fat content or high protein content and 5 soybean plants with low fat content or low protein content |
|  | Microdissection or macrodissection | macrodissection |
|  | Processing procedure | No treatment |
|  | If frozen, how and how quickly? | No |
|  | if fixed, with what and how quickly? | No |
|  | Sample storage conditions and duration (especially for FFPEb samples) | No (we all pick and extract RNA directly) |
| Nucleic acid extraction | Procedure and/or instrumentation | We utilize an RNA extraction kit (EasyPure® Plant RNA Kit) |
|  | Name of kit and details of any modifications | No |
|  | Details of DNase or RNase treatment | DNase I (70μL Reaction Buffer + 10μL DNaseI) |
|  | Contamination assessment (DNA or RNA) | It is generally considered acceptable to have protein or other organic contamination in RNA, and when OD<1.8, protein or phenolic substances remain in the solution. When OD>2.2, the RNA has been hydrolyzed to mononucleic acid. Generally, if the OD is between 1.9 and 2.0, the RNA purity is already high. |
|  | Nucleic acid quantification | 400ng/μL |
|  | Instrument and method | Eppendorf Centrifuge 5810R; Vortex Oscillator DG-800 |
|  | RNA integrity: method/instrument | Agarose gel electrophoresis |
|  | RIN/RQI or Cq of 3_x005f_x005f and 5 transcripts | No |
|  | Inhibition testing (Cq dilutions, spike, or other) | No |
| Reverse transcription | Complete reaction conditions | 25℃ 5min; 42℃ 15min; 85℃ 5min; 4℃ storage |
|  | Amount of RNA and reaction volume | 2.5μl RNA; 20μl system reaction |
|  | Priming oligonucleotide (if using GSP) and concentration | No |
|  | Reverse transcriptase and concentration | SureScript RTase Mix |
|  | Temperature and time | 42℃;15min |
| qPCR target information | Gene symbol | *Glyma.09G158100; Glyma.09G158200; Glyma.12G180200* |
|  | Sequence accession number | XM_003534014.4；XM_003534013.5；XM_003540164.4 |
|  | Amplicon length | 738; 1665; 1053 |
|  | In silico specificity screen (BLAST, and so on) | Yes |
|  | Location of each primer by exon or intron (if applicable) | exon |
|  | What splice variants are targeted? | No |
| qPCR oligonucleotides | Primer sequences | Supplementary 3 |
|  | Location and identity of any modifications | No |
| qPCR protocol | Complete reaction conditions | Two-step method  Pre-denaturation 95℃ 30s Denaturation 95°C 10s  Annealing and extension 60°C 30s |
|  | Reaction volume and amount of cDNA/DNA | 20 μL reaction system; 2 μL |
|  | Primer, (probe), Mg2_x005f_x005f_x005f_x005f_x0001_, and dNTP concentrations | Primer 2μL; 5×BlazeTaq qPCR Mix 4μL |
|  | Polymerase identity and concentration | 5×BlazeTaq qPCR Mix 4μL |
|  | Buffer/kit identity and manufacturer | No |
|  | Additives (SYBR Green I, DMSO, and so forth) | SYBR Green I |
|  | Complete thermocycling parameters | Cycle 1 pre-denaturation 95℃ 30s 40 cycles Denaturation 95℃ 10s  Cycle 40 annealing and extension 60°C 30s |
|  | Manufacturer of qPCR instrument | Agilent Tech nologies Stratagene Mx3000P |
| qPCR validation | Specificity (gel, sequence, melt, or digest) | We assessed the specificity of the amplification products by melting curve analysis. The amplification products showed a single peak, demonstrating high specificity |
|  | For SYBR Green I, Cq of the NTC | SYBR Green I |
|  | Calibration curves with slope and y intercept | Since the standard curve is more inclined to absolute quantification, we did the experiment for relative quantification |
|  | PCR efficiency calculated from slope |  |
|  | r2 of calibration curve |  |
|  | Linear dynamic range |  |
|  | Cq variation at LOD | 30 |
|  | Evidence for LOD | No |
|  | If multiplex, efficiency and LOD of each assay | Because we use a dye method, not a probe method, there is no multiple testing |
| Data analysis | qPCR analysis program (source, version) | EXACL2019 |
|  | Method of Cq determination | 2-ΔΔCt |
|  | Outlier identification and disposition | No |
|  | Results for NTCs | The fluorescence signal of the NTC is very low, which indicates no potential contamination or false positive results during the experiment. |
|  | Justification of number and choice of reference genes | Β-Actin Endogeneity |
|  | Description of normalization method | Internal Reference Gene Method |
|  | Number and stage (reverse transcription or qPCR) of technical replicates | Three biological replicates were taken in the qPCR experiment |
|  | Repeatability (intraassay variation) | We measure experimental reproducibility and error by calculating the standard deviation |
|  | Statistical methods for results significance | One-way ANOVA |
|  | Software (source, version) | Graphpad Prism 9.5.0(https://www.graphpad-prism.cn/） |
