# Supplementary material for: Genome-wide association study and high-quality gene mining related to soybean protein and fat
Source: BMC Genomics. 2023 Oct 7;24:596. doi: 10.1186/s12864-023-09687-6 (PMC10559447; doi:10.1186/s12864-023-09687-6)
Supplement: Supplementary file 5 — Additional file 5: Table S5. Three-year phenotypic data on fat and protein from 292 soybean germplasm resources. [file 12864_2023_9687_MOESM5_ESM.docx]

**Table S5.**Three-year phenotypic data on fat and protein from 292 soybean germplasm resources.

| Line | fat2019(%) | protein2019(%) | fat2020(%) | protein2020(%) | fat2021(%) | protein2021(%) |
| --- | --- | --- | --- | --- | --- | --- |
| K001 | 20.36 | 39.82 | 20.19 | 39.66 | 19.81 | 41.26 |
| K002 | 25.85 | 32.84 | 22.64 | 35.09 | 20.28 | 37.32 |
| K003 | 19.78 | 39.06 | 18.38 | 41.75 | 18.51 | 41.45 |
| K004 | 18.28 | 40.47 | 19.78 | 42.38 | 19.83 | 40.36 |
| K005 | 21.16 | 38.95 | 18.32 | 40.61 | 20.93 | 37.55 |
| K006 | 22.89 | 37.61 | 19.65 | 36.38 | 20.48 | 37.39 |
| K007 | 16.4 | 45.83 | 15.25 | 44.3 | 16.01 | 46.63 |
| K008 | 16.52 | 45.54 | 20.48 | 38.59 | 20.02 | 36.23 |
| K009 | 17.32 | 44.57 | 17.4 | 46.16 | 17.87 | 44.68 |
| K010 | 15.47 | 46.33 | 17.79 | 46.46 | 17.5 | 46.48 |
| K011 | 19.48 | 40.32 | 18.75 | 39.4 | 18.12 | 44.06 |
| K012 | 19.76 | 45.11 | 18.13 | 44.03 | 18.93 | 44.7 |
| K013 | 17.83 | 43.67 | 19.28 | 42.71 | 19.24 | 44.39 |
| K014 | 16.03 | 44.96 | 15.69 | 45.43 | 16.2 | 46.15 |
| K015 | 16.83 | 42.99 | 16.58 | 44.04 | 17.03 | 43.12 |
| K016 | 17.08 | 45.03 | 18.07 | 42.83 | 17.67 | 43.76 |
| K017 | 17.72 | 45.9 | 17.66 | 45.71 | 16.3 | 43.21 |
| K018 | 24.2 | 35.89 | 22.8 | 34.66 | 21.67 | 36.58 |
| K019 | 20.72 | 37.02 | 20.54 | 34.26 | 21.24 | 35.32 |
| K020 | 20.23 | 38.73 | 21.16 | 36.18 | 20.16 | 37.07 |
| K021 | 19.48 | 39.36 | 18.4 | 40.66 | 16.42 | 40.54 |
| K022 | 20.39 | 38.39 | 20.44 | 40.46 | 20.17 | 39.49 |
| K023 | 18.01 | 41.38 | 16.81 | 44.5 | 17.12 | 43.39 |
| K024 | 19.01 | 40.58 | 19.53 | 41.26 | 18.93 | 37.69 |
| K025 | 19.33 | 40.31 | 18.69 | 40.28 | 19.46 | 40.14 |
| K026 | 18.77 | 39.61 | 22.6 | 38.75 | 20.09 | 38.25 |
| K027 | 20.58 | 36.69 | 21.99 | 37.28 | 20.11 | 38.75 |
| K028 | 17.21 | 40.48 | 16.41 | 44.16 | 16.22 | 45.83 |
| K029 | 16.27 | 41.27 | 15.16 | 42.02 | 15.87 | 43.67 |
| K030 | 22.08 | 40.13 | 20.68 | 40.07 | 23.59 | 39.16 |
| K031 | 18.8 | 41.42 | 20.03 | 39.27 | 21 | 39.01 |
| K032 | 17.27 | 41.79 | 19.02 | 39.58 | 18.4 | 40.08 |
| K033 | 20.36 | 36.88 | 21.21 | 39.12 | 20.81 | 37.71 |
| K034 | 22.32 | 34.59 | 18.76 | 37.99 | 20.35 | 39.5 |
| K035 | 17.63 | 38.75 | 17.4 | 43.21 | 16.36 | 42.73 |
| K036 | 17.61 | 44.93 | 17.78 | 44.18 | 16.9 | 47.11 |
| K037 | 20.67 | 38.74 | 20.59 | 37.96 | 21.73 | 36.92 |
| K038 | 19.09 | 41.48 | 17.39 | 44.71 | 19.23 | 40.23 |
| K039 | 18.60 | 42.04 | 16.84 | 44.34 | 16.45 | 43.53 |
| K040 | 21.41 | 39.84 | 22.15 | 39.15 | 21.61 | 38.11 |
| K041 | 18.02 | 44.46 | 17.76 | 44.55 | 17.46 | 44.53 |
| K042 | 20.43 | 39.17 | 20.72 | 39.13 | 19.82 | 38.5 |
| K043 | 21.03 | 38.18 | 19.72 | 41.23 | 18.56 | 40.56 |
| K044 | 20.95 | 40.1 | 23.78 | 36.51 | 22.12 | 37.8 |
| K045 | 20.07 | 41.17 | 19.22 | 40.97 | 20.61 | 39.78 |
| K046 | 19.84 | 39.32 | 19.63 | 40.20 | 19.08 | 39.17 |
| K047 | 20.19 | 39.81 | 19.25 | 38.93 | 19.89 | 40.30 |
| K048 | 14.98 | 44.37 | 14.88 | 46.95 | 15.49 | 47.64 |
| K049 | 16.78 | 44.02 | 16.93 | 41.7 | 16.2 | 43.85 |
| K050 | 20.91 | 35.19 | 20.24 | 38.17 | 20.11 | 38.33 |
| K051 | 17.13 | 42.26 | 17.91 | 43.53 | 17.96 | 43.8 |
| K052 | 19.6 | 40.76 | 17.39 | 43.81 | 18.13 | 39.87 |
| K053 | 21.09 | 39.52 | 21.2 | 36.29 | 20.48 | 40.2 |
| K054 | 20.65 | 38.54 | 22.69 | 35.31 | 24 | 35.18 |
| K055 | 21.26 | 37.4 | 20.52 | 42.15 | 20.55 | 41.5 |
| K056 | 18.25 | 42.9 | 18.51 | 41.45 | 18.75 | 42.48 |
| K057 | 20.41 | 40.87 | 20.05 | 42.45 | 19.40 | 42.72 |
| K058 | 21.38 | 36.25 | 21.2 | 37.93 | 20.58 | 35.02 |
| K059 | 22.07 | 40.84 | 24.61 | 38.07 | 21.01 | 37.72 |
| K060 | 20.37 | 37.01 | 18.73 | 40.15 | 19.07 | 38.86 |
| K061 | 22.64 | 37.55 | 22.98 | 38.11 | 23.01 | 37.23 |
| K062 | 22.22 | 40.55 | 21.86 | 41.03 | 21.95 | 38.78 |
| K063 | 21.02 | 39.87 | 18.27 | 43.74 | 18.45 | 43.74 |
| K064 | 19.03 | 39.44 | 20.01 | 41.53 | 18.83 | 39.1 |
| K065 | 18.97 | 40.98 | 17.23 | 41.68 | 18.38 | 41.47 |
| K066 | 23.01 | 37.26 | 23.30 | 36.12 | 23.52 | 35.79 |
| K067 | 16.41 | 41.16 | 17.72 | 39.7 | 17.48 | 37.99 |
| K068 | 17.91 | 42.28 | 18.53 | 44.55 | 17.13 | 39.11 |
| K069 | 17.14 | 43.54 | 18.9 | 41.19 | 16.87 | 44.87 |
| K070 | 20.15 | 40.98 | 19.08 | 43.25 | 18.91 | 42.77 |
| K071 | 19.66 | 36.27 | 17.37 | 38.09 | 18.40 | 37.06 |
| K072 | 17.27 | 44.04 | 20.55 | 40.51 | 21 | 38.9 |
| K073 | 21.51 | 38.1 | 21.85 | 40.89 | 22.24 | 38.04 |
| K074 | 18.17 | 40.2 | 16.38 | 41.98 | 16.67 | 43.48 |
| K075 | 20.17 | 41.38 | 21.29 | 38.11 | 20.71 | 40.85 |
| K076 | 17.86 | 41.34 | 21.21 | 40.48 | 21.57 | 38.84 |
| K077 | 21.83 | 37.43 | 22.76 | 38.21 | 21.74 | 40.85 |
| K078 | 19.08 | 41.23 | 18.41 | 41.12 | 19.56 | 40.73 |
| K079 | 20.23 | 40.04 | 18.42 | 41.66 | 18.27 | 41.54 |
| K080 | 15.55 | 41.9 | 22.55 | 38.5 | 16.91 | 43.82 |
| K081 | 20.17 | 42.10 | 23.65 | 38.19 | 22.63 | 37.46 |
| K082 | 19.84 | 41.04 | 16.25 | 43.24 | 16.29 | 42.95 |
| K083 | 20.69 | 37.93 | 22.28 | 37.88 | 22.07 | 37.47 |
| K084 | 20.25 | 43.42 | 21.45 | 37.98 | 22.82 | 38.61 |
| K085 | 22.09 | 39.81 | 21.58 | 35.38 | 20.89 | 35.74 |
| K086 | 19.53 | 44.25 | 20.40 | 37.31 | 20.66 | 36.28 |
| K087 | 20.26 | 42.42 | 19.16 | 39.32 | 20.88 | 37.77 |
| K088 | 21.67 | 36.82 | 20.37 | 40.18 | 18.44 | 41.34 |
| K089 | 18.8 | 42.09 | 19.34 | 41.07 | 19.81 | 41.05 |
| K090 | 18.69 | 41.75 | 22.44 | 36.55 | 22.31 | 37.42 |
| K091 | 17.18 | 44.13 | 20.72 | 40.09 | 19.3 | 37.94 |
| K092 | 20.14 | 40.33 | 21.00 | 38.82 | 19.79 | 39.2 |
| K093 | 16.23 | 39.03 | 21.29 | 42.17 | 21.92 | 39.05 |
| K094 | 21.61 | 38.88 | 20.55 | 35.37 | 21.04 | 35.46 |
| K095 | 23.79 | 37.86 | 22.99 | 40.59 | 22.65 | 39.28 |
| K096 | 22.73 | 43.82 | 21.83 | 41.71 | 23.39 | 39.63 |
| K097 | 22.37 | 43.92 | 19.72 | 41.49 | 18.43 | 43.23 |
| K098 | 25.24 | 35.3 | 24.39 | 41.71 | 22.8 | 40.62 |
| K099 | 15.09 | 46.18 | 18.21 | 36.67 | 21.9 | 35.51 |
| K100 | 17.19 | 44.53 | 20.03 | 39.06 | 20.34 | 38.75 |
| K101 | 20.07 | 43.62 | 22.84 | 36.06 | 22.47 | 36.69 |
| K102 | 20.85 | 40.5 | 18.58 | 42.41 | 18.97 | 41.53 |
| K103 | 21.71 | 40.34 | 23.3 | 37.32 | 23.46 | 36.88 |
| K104 | 19.29 | 41.26 | 19.83 | 43.47 | 18.02 | 45.08 |
| K105 | 20.10 | 42.36 | 22.99 | 36.64 | 23.04 | 36.17 |
| K106 | 18.88 | 43.83 | 20.09 | 39.57 | 18.88 | 41.82 |
| K107 | 21.85 | 40.63 | 21.36 | 38.36 | 22.72 | 36.35 |
| K108 | 21.35 | 38.86 | 23.09 | 36.3 | 23.69 | 35.16 |
| K109 | 19.31 | 41.29 | 17.97 | 43.52 | 17.41 | 42.77 |
| K110 | 19.56 | 42.51 | 18.6 | 38.95 | 18.39 | 39.53 |
| K111 | 18.46 | 42.55 | 18.39 | 42.24 | 18.42 | 40.63 |
| K112 | 21.29 | 37.94 | 21.81 | 39.4 | 23.51 | 38.71 |
| K113 | 17.2 | 42.17 | 19.44 | 42.71 | 19.26 | 42.03 |
| K114 | 16.97 | 45.58 | 20.66 | 36.24 | 21.94 | 35.26 |
| K115 | 19.58 | 42.77 | 21.03 | 40.5 | 20.8 | 38.93 |
| K116 | 17.07 | 45.63 | 20.4 | 39.33 | 20.76 | 38.3 |
| K117 | 21.16 | 39.22 | 19.59 | 41.63 | 21.08 | 37.59 |
| K118 | 18.06 | 44.24 | 19.78 | 40.1 | 20.2 | 39.4 |
| K119 | 17.05 | 44.1 | 21.79 | 39.89 | 21.36 | 38.69 |
| K120 | 20.06 | 38.12 | 19.94 | 39.23 | 19.97 | 39.8 |
| K121 | 20.35 | 41.45 | 22.63 | 37.05 | 22.13 | 38.46 |
| K122 | 18.59 | 41.53 | 19.86 | 39.82 | 20.09 | 41.71 |
| K123 | 19.45 | 41.88 | 17.04 | 43.27 | 19.24 | 41.99 |
| K124 | 18.42 | 43.39 | 18.4 | 41.68 | 19.53 | 40.49 |
| K125 | 19.67 | 43.15 | 20.24 | 38.09 | 20.22 | 36.82 |
| K126 | 20.79 | 40.1 | 18.44 | 40.64 | 18.6 | 42.37 |
| K127 | 20.88 | 38.57 | 20.49 | 37.47 | 20.66 | 36.98 |
| K128 | 18.47 | 41.36 | 20.95 | 39.03 | 19.77 | 38.59 |
| K129 | 19.61 | 41.32 | 20.38 | 41.3 | 20.5 | 41.19 |
| K130 | 18.19 | 41.5 | 16.67 | 45.95 | 16.96 | 45.96 |
| K131 | 18.14 | 44.89 | 18.33 | 42.44 | 18.15 | 42.48 |
| K132 | 18.62 | 44.48 | 22.17 | 36.86 | 21.95 | 34.55 |
| K133 | 18.49 | 44.6 | 20.23 | 36.88 | 20.47 | 34.79 |
| K134 | 19.79 | 41.19 | 20.78 | 41.15 | 21.15 | 39.57 |
| K135 | 17.67 | 44.56 | 19.05 | 39.71 | 18.07 | 40.18 |
| K136 | 18.86 | 42.14 | 18.94 | 41.73 | 20.54 | 42.65 |
| K137 | 21.07 | 40.62 | 20.24 | 40.2 | 20.36 | 47.4 |
| K138 | 22.23 | 40.61 | 21.76 | 38.69 | 20.27 | 40.84 |
| K139 | 19.62 | 42.75 | 20.09 | 39.58 | 20.26 | 39.07 |
| K140 | 18.83 | 41.75 | 19.25 | 37.01 | 20.93 | 37.1 |
| K141 | 16.19 | 40.14 | 21.26 | 36.06 | 21.62 | 37.63 |
| K142 | 17.17 | 44.16 | 17.49 | 42.62 | 19.52 | 41.98 |
| K143 | 17.47 | 42.57 | 19.91 | 40.28 | 23.78 | 37.43 |
| K144 | 18.99 | 40.58 | 21 | 36.59 | 21.43 | 39.54 |
| K145 | 18.66 | 38.86 | 19.43 | 39.5 | 19.61 | 39.71 |
| K146 | 20.53 | 40.94 | 22.42 | 36.24 | 22.11 | 36.12 |
| K147 | 15.69 | 41.1 | 19.81 | 38.88 | 22.28 | 37.03 |
| K148 | 16.03 | 39.31 | 19.8 | 42.21 | 20.03 | 41.27 |
| K149 | 17.93 | 45.95 | 20.55 | 37.34 | 18.97 | 38.28 |
| K150 | 17.72 | 43.99 | 20.39 | 39.63 | 20.23 | 38.24 |
| K151 | 19.14 | 39.65 | 18.62 | 41.62 | 16.56 | 41.33 |
| K152 | 17.96 | 44.93 | 17.94 | 41.46 | 17.43 | 41.35 |
| K153 | 20.58 | 38.98 | 19.48 | 41.9 | 19.33 | 40.62 |
| K154 | 21.4 | 40.93 | 21.96 | 38.2 | 21.46 | 40.12 |
| K155 | 18.17 | 43.31 | 19.31 | 42.6 | 17.19 | 44.13 |
| K156 | 19.9 | 40.4 | 20.52 | 40.19 | 20.39 | 39.23 |
| K157 | 17.96 | 44.13 | 19.04 | 43.46 | 19.91 | 42.32 |
| K158 | 19.16 | 40.94 | 19.94 | 40.65 | 21.34 | 38.48 |
| K159 | 21.27 | 39.6 | 23.39 | 35.37 | 23.84 | 34.90 |
| K160 | 20.83 | 42.11 | 20.95 | 40.89 | 20.82 | 39.77 |
| K161 | 20.6 | 40.53 | 20.66 | 38.02 | 20.53 | 39.14 |
| K162 | 19.29 | 44.29 | 20.17 | 42.17 | 19.98 | 38.25 |
| K163 | 19.19 | 42.23 | 22.4 | 39.35 | 21.77 | 38 |
| K164 | 17.63 | 39.78 | 21.07 | 41.14 | 20.24 | 42.93 |
| K165 | 19.79 | 43.31 | 19.36 | 45.39 | 19.32 | 42 |
| K166 | 19.33 | 44.81 | 21.33 | 40.12 | 20.66 | 39.94 |
| K167 | 18.18 | 42.69 | 19.2 | 39.12 | 19.05 | 40.58 |
| K168 | 19.81 | 44.46 | 20.09 | 41.32 | 20.01 | 39.26 |
| K169 | 19.96 | 41.81 | 19.54 | 38.3 | 20.67 | 37.82 |
| K170 | 22.11 | 40.21 | 21.42 | 36.39 | 21.18 | 37.39 |
| K171 | 20.64 | 40.77 | 19.6 | 41.21 | 21.26 | 37.84 |
| K172 | 15.81 | 39.3 | 18.27 | 42.48 | 18.59 | 41.64 |
| K173 | 18.2 | 42.04 | 21.36 | 37.08 | 20.83 | 35.66 |
| K174 | 18.44 | 42.05 | 24.48 | 38.34 | 21.65 | 39.61 |
| K175 | 18.91 | 41.99 | 20.62 | 40.92 | 22.35 | 38.04 |
| K176 | 19.62 | 42.8 | 19.95 | 40.48 | 20.14 | 39.2 |
| K177 | 20.28 | 40.47 | 17.54 | 44.58 | 17.33 | 45.54 |
| K178 | 18.34 | 44.48 | 19.1 | 38.71 | 20.18 | 39.97 |
| K179 | 21.32 | 39.8 | 22.34 | 41.16 | 22.2 | 35.2 |
| K180 | 18.48 | 43.9 | 19.27 | 40.06 | 19.83 | 37.95 |
| K181 | 20.12 | 41.95 | 18.01 | 42.06 | 18.19 | 40.94 |
| K182 | 16.8 | 46.74 | 16.6 | 45.56 | 16.46 | 44.53 |
| K183 | 15.78 | 42.71 | 22.37 | 35.36 | 20.98 | 36.33 |
| K184 | 16.88 | 40.9 | 20.3 | 40.01 | 21.93 | 38.34 |
| K185 | 20.96 | 38.26 | 21.57 | 38.76 | 21.07 | 37.99 |
| K186 | 17.83 | 40.91 | 20.20 | 38.18 | 19.27 | 38.33 |
| K187 | 20.87 | 40.09 | 20.76 | 40.3 | 21.18 | 37.73 |
| K188 | 21.72 | 40.23 | 17.76 | 45.7 | 19.18 | 43.9 |
| K189 | 22.21 | 40.35 | 19.06 | 42.81 | 20.23 | 41.62 |
| K190 | 17.74 | 42.04 | 16.22 | 45.75 | 18.24 | 41.25 |
| K191 | 19.74 | 43.13 | 17.67 | 39.55 | 18.33 | 37.59 |
| K192 | 20.96 | 40.77 | 21.23 | 41.44 | 22.35 | 37.74 |
| K193 | 23.13 | 38.19 | 20.17 | 41.82 | 22.15 | 38.65 |
| K194 | 20.93 | 37.88 | 20.75 | 36.9 | 21.41 | 40.3 |
| K195 | 23.46 | 36.16 | 19.52 | 37.79 | 21.21 | 36.17 |
| K196 | 19.75 | 41.02 | 19.56 | 37.95 | 18.69 | 41.31 |
| K197 | 17.77 | 40.53 | 17.61 | 41.78 | 17.62 | 41.27 |
| K198 | 22.47 | 37.58 | 21.6 | 37.69 | 20.32 | 36.58 |
| K199 | 21.58 | 40.4 | 19.9 | 38.81 | 20.54 | 37.48 |
| K200 | 18.35 | 39.89 | 18.76 | 41.26 | 18.24 | 44.85 |
| K201 | 22.86 | 37.28 | 21.38 | 38.37 | 19.31 | 40.3 |
| K202 | 20.93 | 38.15 | 19.7 | 40.02 | 22.68 | 40.03 |
| K203 | 20.4 | 41.13 | 20.15 | 43.05 | 20.05 | 41.62 |
| K204 | 19.8 | 40.85 | 19.17 | 42.32 | 19.71 | 41.94 |
| K205 | 18.56 | 40.58 | 20.01 | 38.98 | 18.71 | 42.35 |
| K206 | 19.88 | 40.44 | 17.7 | 44.82 | 18.47 | 44.52 |
| K207 | 20.74 | 41.66 | 20.74 | 39.98 | 19.50 | 40.40 |
| K208 | 21.18 | 37.09 | 21.74 | 36.34 | 21.63 | 36.43 |
| K209 | 20.41 | 35.58 | 21.38 | 37.56 | 22.21 | 34.46 |
| K210 | 22.59 | 35.51 | 20.27 | 39.03 | 20.93 | 37.18 |
| K211 | 20.3 | 36.34 | 19.2 | 38.06 | 18.73 | 40.23 |
| K212 | 22.86 | 37.61 | 19.96 | 42.82 | 19.61 | 41.72 |
| K213 | 23.38 | 36.13 | 20.12 | 37.15 | 20.13 | 38.79 |
| K214 | 18.22 | 41.99 | 18.13 | 40.76 | 18.26 | 45.23 |
| K215 | 23.15 | 37.84 | 21.71 | 39.86 | 22.29 | 38.78 |
| K216 | 22.74 | 39.68 | 20.91 | 40.42 | 21.86 | 39.67 |
| K217 | 21.78 | 39.23 | 20.59 | 41.85 | 20.26 | 42.55 |
| K218 | 18.85 | 40.59 | 18.82 | 38.55 | 16.51 | 43.43 |
| K219 | 21.32 | 38.1 | 19.01 | 39.99 | 18.32 | 40.97 |
| K220 | 20.73 | 37.74 | 20.41 | 40.3 | 19.32 | 39.25 |
| K221 | 20.58 | 37.88 | 20.27 | 40.96 | 18.89 | 42.38 |
| K222 | 22.59 | 36.79 | 19.45 | 44.46 | 21.64 | 37.72 |
| K223 | 20.34 | 40.59 | 21.64 | 38.45 | 20.3 | 40.2 |
| K224 | 20.65 | 37.18 | 16.81 | 42.41 | 21.15 | 36.86 |
| K225 | 23.52 | 37.22 | 22.51 | 38.75 | 22.75 | 35.01 |
| K226 | 25.86 | 31.13 | 18.72 | 37.41 | 21.56 | 35.63 |
| K227 | 20.17 | 39.41 | 18.38 | 39.3 | 18.29 | 39.76 |
| K228 | 19.94 | 37.97 | 18.28 | 42.09 | 19.34 | 39.97 |
| K229 | 18.53 | 39.17 | 18.03 | 42.37 | 19.06 | 40.24 |
| K230 | 18.49 | 41.05 | 18.29 | 42.48 | 19.81 | 39.15 |
| K231 | 20.83 | 38.17 | 17.63 | 42.7 | 20.86 | 37.27 |
| K232 | 22.51 | 35.47 | 20.94 | 37.97 | 21.45 | 33.09 |
| K233 | 20.68 | 38.56 | 19.9 | 40.16 | 21.49 | 37.43 |
| K234 | 21.41 | 39.93 | 22.74 | 36.6 | 22.78 | 35.14 |
| K235 | 21.3 | 37.79 | 19.55 | 38.14 | 20.48 | 39.39 |
| K236 | 21.09 | 36.17 | 18.98 | 44.83 | 21.9 | 35.35 |
| K237 | 18.66 | 41.82 | 17.85 | 41.21 | 18.32 | 41.49 |
| K238 | 22.46 | 38.34 | 22.3 | 41.15 | 19.46 | 42.77 |
| K239 | 20.44 | 39.05 | 19.48 | 37.77 | 18.62 | 40.49 |
| K240 | 18.16 | 41.82 | 17.66 | 44.62 | 18.32 | 43.93 |
| K241 | 20.05 | 41.03 | 17.49 | 45.9 | 19.87 | 40.59 |
| K242 | 19.75 | 40.24 | 20.47 | 36.83 | 19.72 | 36.48 |
| K243 | 20.86 | 41.15 | 18.15 | 41.81 | 19.66 | 41.32 |
| K244 | 18.94 | 44.62 | 19.81 | 42.33 | 18.55 | 41.56 |
| K245 | 21.94 | 35.81 | 20.77 | 39.47 | 17.59 | 43.29 |
| K246 | 20.35 | 39.54 | 18.16 | 40.18 | 19.49 | 41.75 |
| K247 | 22.63 | 36.91 | 20.36 | 38.09 | 20.42 | 37.78 |
| K248 | 19.07 | 38.95 | 19.09 | 42.86 | 19.58 | 42.04 |
| K249 | 20.78 | 38.37 | 21.91 | 41.37 | 20.81 | 42.47 |
| K250 | 21.15 | 36.06 | 20.14 | 40.97 | 18.75 | 40.01 |
| K251 | 23.31 | 35.05 | 18.99 | 40.49 | 20.52 | 38.14 |
| K252 | 20.73 | 37.1 | 18.34 | 35.08 | 18.68 | 37.3 |
| K253 | 21.35 | 37.84 | 18.14 | 42.69 | 19.77 | 42.35 |
| K254 | 19.36 | 38.28 | 20.76 | 36.8 | 21.23 | 39.01 |
| K255 | 21.57 | 39.43 | 21.23 | 39.01 | 20.31 | 43.26 |
| K256 | 24.29 | 36.83 | 20.4 | 41.59 | 20.39 | 40.47 |
| K257 | 24.97 | 36.35 | 22.21 | 32.08 | 21.91 | 37.28 |
| K258 | 23.84 | 39.24 | 22.3 | 32.64 | 22.84 | 36 |
| K259 | 24.39 | 36.69 | 21.26 | 36.56 | 24.28 | 35.45 |
| K260 | 21.98 | 39.47 | 22.06 | 36.98 | 22.07 | 35.36 |
| K261 | 22.34 | 36.25 | 23.23 | 39.95 | 22.4 | 39.02 |
| K262 | 23.55 | 36.18 | 24.51 | 37.92 | 24.6 | 38.39 |
| K263 | 20.75 | 38.63 | 20.95 | 41.57 | 20.73 | 41.45 |
| K264 | 21.15 | 36.43 | 22.34 | 39.51 | 22.35 | 38.54 |
| K265 | 22.2 | 36.83 | 22.02 | 40.69 | 22.44 | 39.54 |
| K266 | 20.97 | 37.58 | 22.16 | 40.85 | 21.39 | 39.57 |
| K267 | 22.47 | 37.72 | 22.52 | 40.16 | 23.17 | 39.35 |
| K268 | 21.81 | 39.34 | 22.54 | 41.69 | 22.06 | 42.03 |
| K269 | 18.13 | 42.61 | 18.63 | 44.59 | 18.72 | 44.64 |
| K270 | 20.87 | 39.09 | 20.61 | 41.39 | 22.2 | 40.69 |
| K271 | 20.91 | 36.85 | 20.68 | 40.24 | 21.07 | 39.54 |
| K272 | 21 | 39.07 | 21.64 | 42.24 | 22.07 | 42.3 |
| K273 | 20.89 | 37.3 | 20.78 | 40.2 | 22 | 39.52 |
| K274 | 23.82 | 34.46 | 25.6 | 37.92 | 23.97 | 37.46 |
| K275 | 22.1 | 35.39 | 22.44 | 38.83 | 23.57 | 38.43 |
| K276 | 22.51 | 35.44 | 22.87 | 39.57 | 22.8 | 38.09 |
| K277 | 20.57 | 42.59 | 20.21 | 44.92 | 20.46 | 45.92 |
| K278 | 20.34 | 37.46 | 20.27 | 40.2 | 20.92 | 40.12 |
| K279 | 21.42 | 35.86 | 21.48 | 39.24 | 22.05 | 39.18 |
| K280 | 20.93 | 36.2 | 21.85 | 39.02 | 21.44 | 37.99 |
| K281 | 24.55 | 33.61 | 24.87 | 35.37 | 24.7 | 35.87 |
| K282 | 20.36 | 39.12 | 20.57 | 41.52 | 21.73 | 40.84 |
| K283 | 22.53 | 35.35 | 23.08 | 39.65 | 23.57 | 37.93 |
| K284 | 20.35 | 41.12 | 20.72 | 43.91 | 20.88 | 43.44 |
| K285 | 20.75 | 36.92 | 21.51 | 40.77 | 20.58 | 39.03 |
| K286 | 19.54 | 40.83 | 20.37 | 43.1 | 20.55 | 43.4 |
| K287 | 20.38 | 41.31 | 21.15 | 44.3 | 21.23 | 43.54 |
| K288 | 19.34 | 40.44 | 20.4 | 43.11 | 20.47 | 42.69 |
| K289 | 21.39 | 39.42 | 20.94 | 43.29 | 21.48 | 39.34 |
| K290 | 18.96 | 44.03 | 17.2 | 43 | 18.11 | 43.61 |
| K291 | 20.76 | 38.96 | 21.28 | 42.08 | 19.81 | 40.2 |
| K292 | 19.84 | 39.43 | 17.88 | 41.49 | 18.52 | 40.31 |
