# Supplementary material for: Genome-wide association study and high-quality gene mining related to soybean protein and fat
Source: BMC Genomics. 2023 Oct 7;24:596. doi: 10.1186/s12864-023-09687-6 (PMC10559447; doi:10.1186/s12864-023-09687-6)
Supplement: Supplementary file 6 — Additional file 6: Table S6. Information on candidate genes related to fat and protein content of soybean. [file 12864_2023_9687_MOESM6_ESM.docx]

**Table S6.** Information on candidate genes related to fat and protein content of soybean.

| Trait | Gene ID | Chr | Start | End | Allel | SNP position | Pvalue | Function annotation |
| --- | --- | --- | --- | --- | --- | --- | --- | --- |
| Fat | *Glyma.12G180200* | Gm_12 | 35527978 | 35530022 | T/C | 35492373 | 4.97E-05 | Molecular Function: hydrolase activity, acting on ester bonds (GO:0016788); |
| Protein | *Glyma.09G158100* | Gm_09 | 39035937 | 39039823 | C/G | 39012959 | 8.4E-4 | Biological Process: ribosomal subunit export from nucleus (GO:0000054);; Biological Process: maturation of 5.8S rRNA (GO:0000460);; Biological Process: maturation of LSU-rRNA (GO:0000470);; Molecular Function: translation initiation factor activity (GO:0003743);; Biological Process: mature ribosome assembly (GO:0042256);; Molecular Function: ribosome binding (GO:0043022);; Molecular Function: ribosomal large subunit binding (GO:0043023);; Biological Process: assembly of large subunit precursor of preribosome (GO:1902626); |
|  | *Glyma.09G158200* | Gm_09 | 39040259 | 39046458 | C/G | 39012959 | 8.5E-4 | Molecular Function: ATP binding (GO:0005524);; Cellular Component: chaperonin-containing T-complex (GO:0005832);; Biological Process: protein folding (GO:0006457);; Molecular Function: unfolded protein binding (GO:0051082); |
